# Single-cell RNA transcriptomic reveal the mechanism of MSC derived small extracellular vesicles against DKD fibrosis

**DOI:** 10.1186/s12951-024-02613-2

**Published:** 2024-06-18

**Authors:** Cheng Ji, Jiahui Zhang, Hui Shi, Binghai Chen, Wenrong Xu, Jianhua Jin, Hui Qian

**Affiliations:** 1 Wujin Institute of Molecular Diagnostics and Precision Cancer Medicine of Jiangsu University, Wujin Hospital Affiliated with Jiangsu University, Chang Zhou, Jiangsu 213004 China; 2 Jiangsu Key Laboratory of Medical Science and Laboratory Medicine, Department of Laboratory Medicine, School of Medicine, Jiangsu University, Zhenjiang, Jiangsu 212013 China; 3 Institute of Translational Medicine, Department of Urology, Jiangsu University, Affiliated Hospital of Jiangsu University, Zhenjiang, Jiangsu 212001 China; 4 NHC Key Laboratory of Medical Embryogenesis and Developmental Molecular Biology, Shanghai Key Laboratory of Embryo and Reproduction Engineering, ShangHai, 200040 China

**Keywords:** Single-cell RNA sequencing, Fibrosis-associated macrophages, Mesangial, Mesenchymal stem cells, Extracellular vesicles, Antifibrosis niche

## Abstract

Diabetic kidney disease (DKD), a chronic kidney disease, is characterized by progressive fibrosis caused due to persistent hyperglycemia. The development of fibrosis in DKD determines the patient prognosis, but no particularly effective treatment. Here, small extracellular vesicles derived from mesenchymal stem cells (MSC-sEV) have been used to treat DKD fibrosis. Single-cell RNA sequencing was used to analyze 27,424 cells of the kidney, we have found that a novel fibrosis-associated TGF-β_1_^+^Arg1^+^ macrophage subpopulation, which expanded and polarized in DKD and was noted to be profibrogenic. Additionally, Actin^+^Col4a5^+^ mesangial cells in DKD differentiated into myofibroblasts. Multilineage ligand-receptor and cell-communication analysis showed that fibrosis-associated macrophages activated the TGF-β_1_/Smad2/3/YAP signal axis, which promotes mesangial fibrosis-like change and accelerates renal fibrosis niche. Subsequently, the transcriptome sequencing and LC-MS/MS analysis indicated that MSC-sEV intervention could restore the levels of the kinase ubiquitin system in DKD and attenuate renal interstitial fibrosis via delivering CK1δ/β-TRCP to mediate YAP ubiquitination degradation in mesangial cells. Our findings demonstrate the unique cellular and molecular mechanisms of MSC-sEV in treating the DKD fibrosis niche at a single-cell level and provide a novel therapeutic strategy for renal fibrosis.

**Supplementary Information:**

The online version contains supplementary material available at 10.1186/s12951-024-02613-2.

## Introduction

According to data reported by the International Diabetes Federation, 425 million people worldwide have diabetes, causing 1 million deaths annually, and the incidence is increasing [1–3]. Iterative kidney injury is the most common cause of progressive fibrosis in diabetic kidney disease (DKD), ultimately resulting in renal fibrosis [4, 5]. DKD is a microvascular complication of diabetes that causes end-stage renal disease (ESRD) [6]. The degree of renal fibrosis predicts the severity and likelihood of adverse patient outcomes [7]. Kidney fibrosis involves a complex interplay between multiple nonparenchymal cell lineages-including renal tubules and immune, endothelial, and mesenchymal cells located in scarred areas, termed the fibrotic niche [8, 9]. So, antifibrotic therapies for patients with chronic kidney disease are urgently required [10–12]. Interestingly, small extracellular vesicles, nanoscale membrane particles derived from mesenchymal stem cells (MSC-sEV) can repair nephrotoxicity and inhibit its fibrosis via delivering proteins and nucleic acids such as 14-3-3 ζ to activate autophagy in our findings [13, 14]. Based progress in our understanding kidney fibrogenesis from findings on rodent models. However, the functions and mechanisms of MSC-sEV involving in the fibrotic niche are not well understood [15]. In this study, we have further analyzed the functional heterogeneity of the renal fibrosis niche and the interaction of cell lineages though single-cell RNA sequencing.

Here, single-cell RNA sequencing (sc-RNA Seq), which revolutionizes our understanding of disease pathogenesis [16–18]. We used single cell sequencing to study the underlying mechanisms of MSC-sEV regulating DKD interstitial fibrosis. We found the changes in renal cell community in rats using the clustering dimension reduction method. The violin atlas marker gene indicates the communities of macrophages and glomerular mesangial cells. The changes in the distribution of renal cell communities after MSC-sEV administration, such as changes in the number of renal intrinsic cells (e.g., macrophages and glomerular mesangial cells). Findings on the kidney transcriptome chip and on communication between cell communities and differential gene enrichment (from differential gene ontology enrichment analysis and KEGG enrichment analysis) are used for personalized analysis and identification. The screening of inflammatory factors released by the recruitment of macrophage aggregation and infiltration is used to analyze the interaction between macrophages and glomerular mesangial cells with the aid of the ligand-receptor database cell communication relationship. A coculture system of macrophages and mesangial cells was established in *vitro*. The changes in levels of the fibrotic markers α-SMA and Collagen I of mesangial cells were observed using confocal microscopy, and the findings revealed that the interaction between macrophages and mesangial cells was involved in the process of DKD fibrosis.

In this study, we found that during the progression of DKD, continuous hyperglycemia stimulates macrophage activation, causing macrophages to secrete chemicals for fibrosis, promoting the transformation of glomerular mesangial cells into myofibroblasts and the progression of renal interstitial fibrosis. Moreover, MSC-sEV transported CK1δ/β-TRCP to promote YAP ubiquitination degradation and alleviate DKD progression. Our findings indicated that MSC-sEV as a versatile delivery system for the kinase and the ubiquitin system provides a novel antifibrotic strategy for DKD.

## Results

### Inhibition activity of MSC-sEV for interstitial fibrosis in the DKD model

**Fig. 1:**
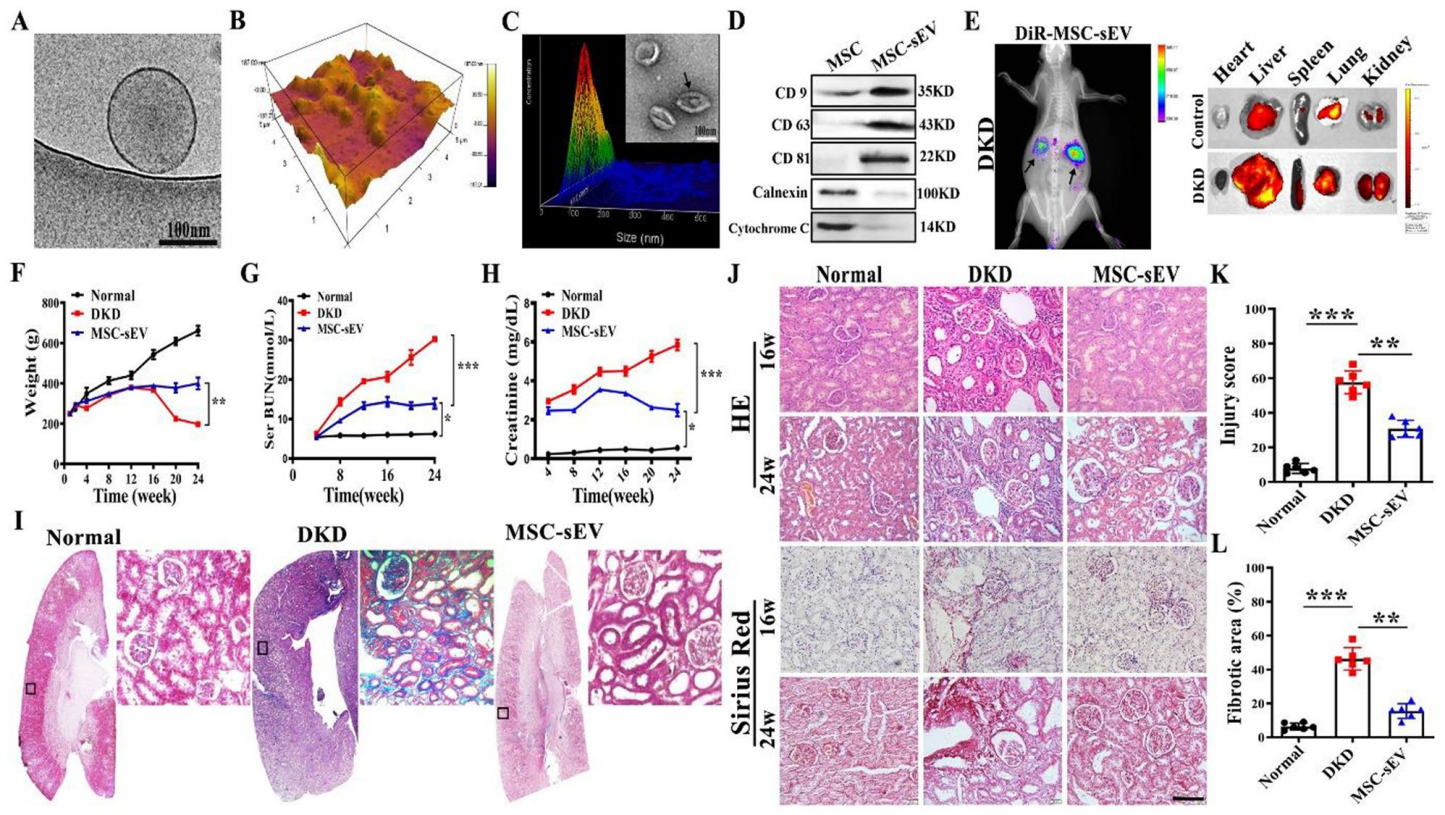
MSC-sEV attenuates interstitial fibrosis in the DKD model. (**A**) Representative images of MSC-sEV detected by Cryo-TEM. Scale bar, 100 nm. (**B**) Three-dimensional spherical structure of AFM for MSC-sEV. (**C**) Size distribution and representative TEM images of MSC-sEV. Scale bar, 100 nm. (**D**) Western blot for positive (CD9, CD63, and CD81) and negative (Calnexin, Cytochrome C) markers in MSC-sEV. (**E**) Imaging of fluorescence intensity of indicated organs at 48 h after CM-DiR labeled MSC-sEV injection detected by IVIS system. (**F**) The body weight of the rats in the Normal, DKD, and MSC-sEV groups monitored for 24 weeks. (**G–H**) Effects of MSC-sEV on serum urea nitrogen and serum creatinine detected by a serum biochemical analyzer (*n* = 10). (**I**) Representative images of Masson trichrome staining on sagittal sections (*n* = 6). The bottom image is a magnified image of the corresponding region. Scale bar, 1 mm (top) and 200 μm (bottom). (**J**) Representative images of H&E and Sirius red staining of kidneys in DKD rats treated with MSC-sEV. Scale bar, 100 μm. (**K**) Quantification of renal injury based on H&E staining (*n* = 6). (**L**) Quantification of renal fibrosis based on Sirius red staining (*n* = 6). * *p* < 0.05, ** *p* < 0.01, *** *p* < 0.001

MSC-sEV isolated per the method of a previous study was identified the biological characteristics of MSC-sEV under a Cryo-transmission electron microscope (Cryo-TEM) and presented cup-like spherical vesicles (Fig. 1A). Atomic force microscope showed that the spherical vesicles had a three-dimensional structure (Fig. 1B). Nanoparticle tracking results showed that the MSC-sEV had a size of 105.2 ± 30.1 nm (mean ± standard deviation) and small microvesicle-like particles (Fig. 1C). The isolated MSC-sEV expressed CD9, CD63, and CD81 but negative for proteins such as calnexin and cytochrome C (Fig. 1D).

To evaluate the therapeutic efficacy of MSC-sEV, we established a rat model of DKD (Fig. S1) and administered MSC-sEV (10 mg/kg) intravenously after streptozotocin (STZ, 35 mg/kg) injury once every 3 days for 6 months (Fig. S2A). MSC-sEV labeling with 1,1-dioctadecyl-3,3,3,3-tetramethylindotricarbocyaineiodide (DIR) was mainly distributed in the liver, with only small amounts in the kidney, in the control rats. In contrast, renal DIR fluorescence significantly increased in the DKD group and peaked 48 h after injection (Fig. 1E and Fig. S2B). Then we infused MSC-CM, MSC-sEV, and human lung fibroblast-sEV (HFL1-sEV) into DKD rats to verify that MSC-sEV is a key component in renal fibrosis repair (Fig. S3). As the figures showed that MSC-sEV ameliorated caused significant weight loss and markedly elevated levels of urea nitrogen and serum creatinine in the DKD group (Fig. 1F-H). Histological analysis of masson trichrome, haematoxylin and eosin (H&E), and sirius red staining of kidney sections at 24 weeks after DKD revealed that MSC-sEV treatment significantly improved renal tubulointerstitial injury, thickening of the glomerular basement membrane, and deposition of a large number of numerous collagen fibers (Fig. 1I and J). A statistical analysis presented that the renal injury score and fibrosis-positive area was increased in the DKD group, while the MSC-sEV intervention significantly reduced the size of the fibrosis-positive area (Fig. 1K and L), and decreased the levels of fibrosis-related proteins (Fig. S4). These results indicated that MSC-sEV ameliorated renal fibrosis, delaying DKD progression.

### The properties of single-cell atlas after MSC-sEV administration in DKD renal

**Fig. 2:**
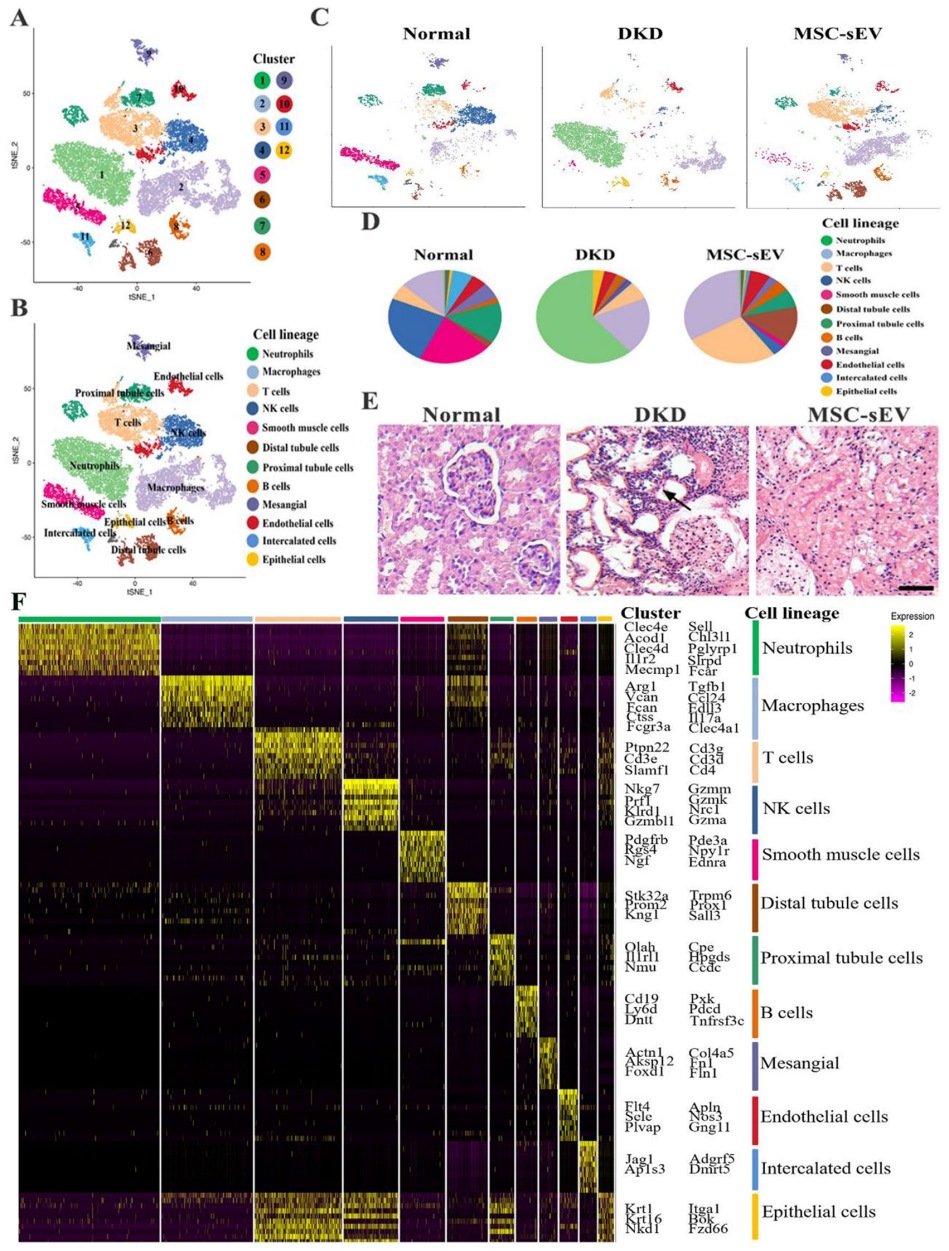
Single-cell atlas and pathology of DKD kidney tissues after MSC-sEV administration. (**A**) Overview: isolation and sc-RNA Seq of DKD renal tissue cells. Clustering 27,424 cells from Normal, DKD, and MSC-sEV groups. Cell lineage inferred from the expression of marker gene signatures. (**B**) Twelve cell clusters and lineages, neutrophils, macrophage, T cells, NK cells, smooth muscle cells, distal tubule cells, proximal tubule cells, B cells, mesangial cells, endothelial cells, intercalated cells, epithelial cells. (**C**) Cell community maps of the three groups and annotation by injury condition. (**D**) Statistical analysis of proportion of renal cell population in Normal, DKD, and MSC-sEV groups. (**E**) H&E staining showed inflammatory cells in DKD renal tissue. Scale bar, 100 μm. (**F**) Heatmap: cluster marker (top, color-coded by cluster) and exemplar genes and lineage annotation labeled (right). Cells present in columnar, genes presented in rows

To verify the feasibility and validity of the MSC-sEV against DKD fibrosis, we used sc-RNA Seq technology to determine the antifibrotic mechanism. We collected kidney tissues from the rats in the normal, DKD, and MSC-sEV groups to prepare single-cell suspensions. Kidney cells were analyzed using bioinformatic analysis (Fig. S5A). In total 27,424 kidney-resident cells from three kidneys fell into 12 populations upon clustering. The data set on kidney tissue cells was annotated using signatures of known lineage markers (Fig. 2A), each containing renal cells from the control, DKD, and MSC-sEV groups across 12 cell clusters and lineages (Fig. 2B and C).

A statistical analysis presented that the number of inflammatory cells such as neutrophils and macrophages in DKD in the treatment groups were significantly higher that of the control group. On the contrary, the number of renal intrinsic cells (tubular epithelial cells, endothelial cells) decreased significantly (Fig. S5). MSC-sEV significantly reduced the number of inflammatory cells, while remaining normal the distribution of cell communities (Fig. 2D and Fig. S6), and the pathological H&E staining of renal tissue confirmed this phenomenon (Fig. 2E). Furthermore, focused on the gene expression of cell communities we found the levels of the 10 most prevalent gene markers in all subpopulations across all cell clusters and lineages in kidney tissues in heat map (Fig. 2F).

These results suggest that MSC-sEV restores the cell cluster changes induced by DKD and inhibits the infiltration of inflammatory cells.

### Interaction between the macrophages and mesangial lineage in DKD fibrotic niche after MSC-sEV administration

**Fig. 3:**
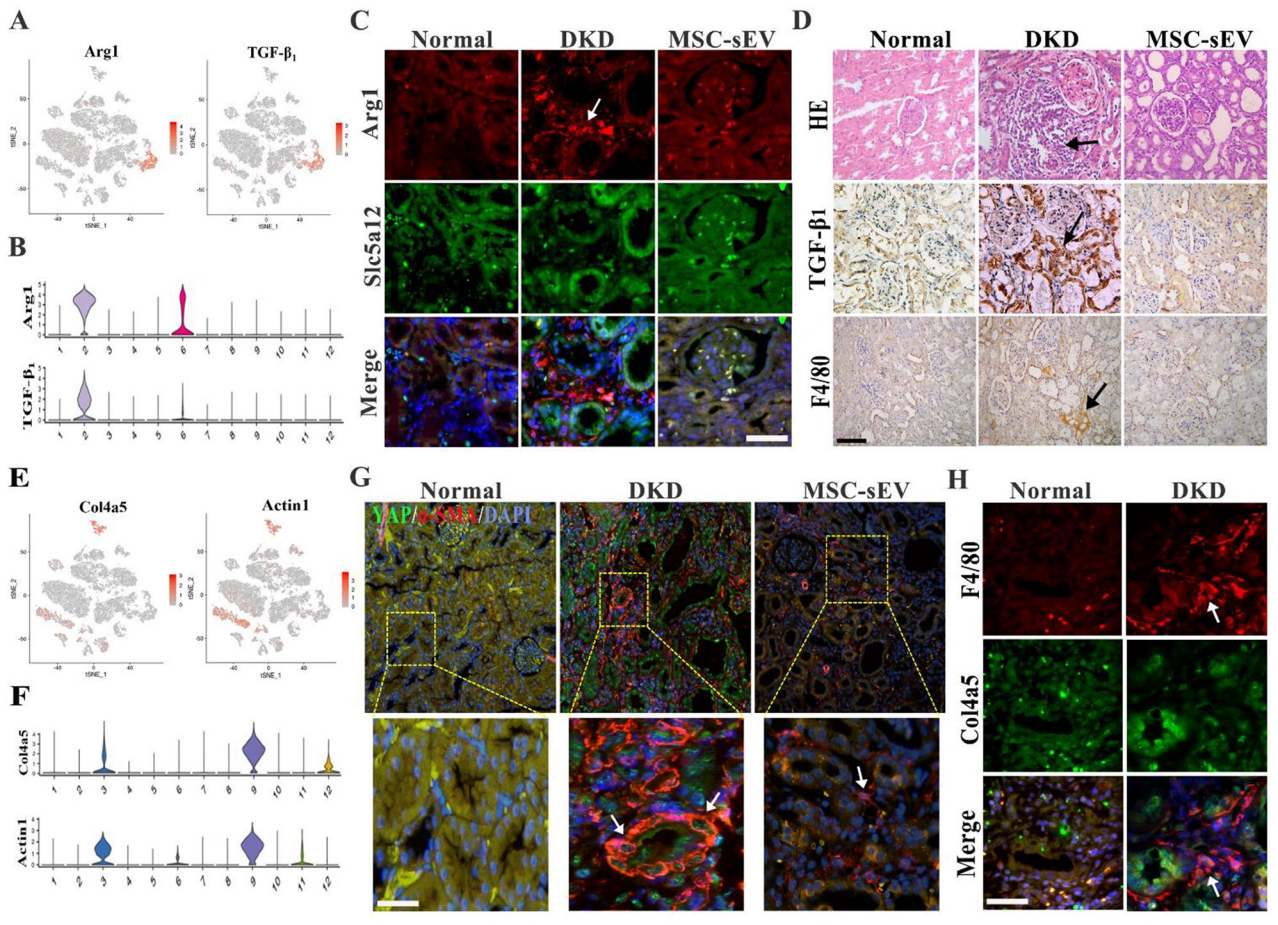
The populations of fibrosis-associated macrophages and interaction with mesangial cells in the fibrotic niche. (**A**) Marker gene expression of FAM in tSNE map. (**B**) Scaled gene expression of FAM cluster markers in DKD. (**C**) Representative immunofluorescence images of FAM in kidney fibrotic niche. top, Arg1 (red), Slc5a12 (green), and DAPI (blue); arrows, Arg1^+^FAM; Scale bar, 50 μm. (**D**) Pathological H&E staining and immunohistochemical staining showed TGF-β_1_ and F4/80-positive FAM in DKD renal tissue. Scale bar, 100 μm. (**E**) Marker gene expression of mesangial cells in tSNE map. (**F**) Scaled gene expression of mesangial cells markers. (**G**) Representative immunofluorescence images of fibrotic mesangial cells in DKD: α-SMA (red), YAP (green), and DAPI (blue), Scale bar, 50 μm. (**H**) Representative immunofluorescence images, kidney fibrotic niche colocalization. Top, F4/80 (red), Col4a5 (green), and DAPI (blue); arrows, F4/80^+^FAM.

Studies have demonstrated that macrophage subpopulations are orchestrated in organ fibrosis progression and regression [19–21]. To explore the exact role of macrophage subsets in DKD renal organ fibrosis, we focused on the sc-RNA Seq analysis results showing the cellularity of the macrophage population increased in the DKD kidneys and macrophages expressed the unique markers of TGF-β_1_ and Arg1 (Fig. 3A and B). Then, confocal graphs showed that the levels of Arg1 labeled-macrophages were significantly increased in *vivo*, and the kidney pathology presented monocyte infiltration in the glomerular area (Fig. 3C). Furthermore, following injury, F4/80 and TGF-β_1_ positive fibrosis-associated macrophages derived from circulating monocytes accumulated in the kidney. These results appeared that macrophage clusters polarized fibrosis-associated macrophage phenotype features in DKD rats (Fig. 3D). After MSC-sEV treatment, the number of TGF-β_1_ and F4/80 positive FAM decreased significantly in DKD (Fig. 3C and D). Confocal microscopy results showed that MSC-sEV could induced the shift the phenotypes of DKD associated macrophages M2 and M1 and inhibitory effect on macrophage-myofibroblast transition cells (Fig. S7 and S8). These results indicate that MSC-sEV inhibit the shift in renal fibrosis-associated macrophage population in DKD to the fibrotic phenotype.

In addition, RNA sequencing results showed significantly reduced levels of DKD innate cells, such as tubular epithelial cells, endothelial cells, and mesangial cells. The mesangial cell clusters expressed the fibrosis-associated markers Actin and Col4a5 (Fig. 3E and F). The fibrosis marker α-SMA was detected in *vivo* by immunofluorescence assay, and the fibrosis-like mesangial cells were found in 24 weeks DKD rats (Fig. 3G). We found that F4/80^+^ FAM colocalized with Col4a5-positive mesangial cells in DKD kidney tissues (Fig. 3H). These results suggest that fibrosis-associated macrophage and myofibroblast transformation of mesangial cells might participate in the renal fibrotic niche.

### Fibrosis-associated macrophages promote mesangial-to-myofibroblast differentiation via the TGF-β_1_/Smad2/3/YAP axis

**Fig. 4:**
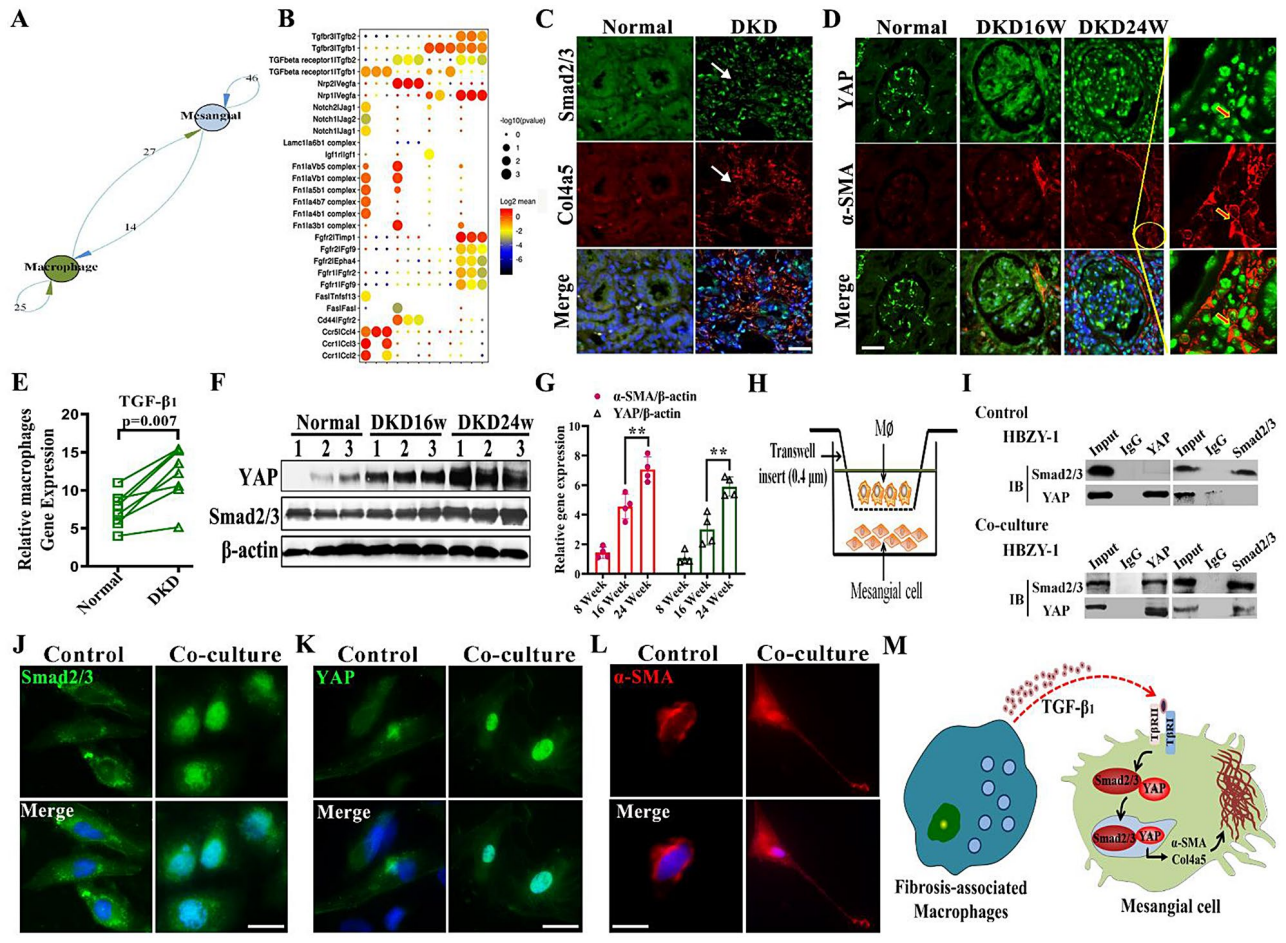
Fibrosis-associated macrophages promote fibrosis-like changes in mesangial cells via TGF-β_1_/Smad2/3/YAP signal axis. (**A**) Ligand-receptor database analysis of fibrosis network between macrophages and mesangial cells. (**B**) Dot plot: ligand-receptor interactions between FAM and mesangial. X-axis, cell populations expressing ligand and receptor; Y-axis, ligand, and cognate receptor; circle size, p value (permutation test); color (red, high; black, low), means of average ligand and receptor expression levels in interacting subpopulations. (**C**) Representative immunofluorescence images of fibrotic niche in DKD. Smad2/3 (green), Col4a5 (red), and DAPI (blue). Scale bar, 50 μm. (**D**) Representative confocal images of mesangial cells in DKD kidney sections. YAP (green), α-SMA (red), and DAPI (blue). Scale bar, 50 μm. (**E**) qRT-PCR assay for TGF-β_1_ in macrophages stimulated by high glucose. (**F**) Western blotting analysis of Smad2/3 and YAP in 16-week and 24-week DKD kidney tissues (*n* = 3). (**G**) Statistical analysis of expressions of α-SMA and YAP. (**H**) Mesangial cell activation assay: co-culture of mesangial cells from uninjured rat kidney and high glucose domesticated macrophage subpopulations (MΦ) from DKD rat kidney. (**I**) Co-immunoprecipitation analysis of Smad2/3 and YAP colocalization in fibrotic-like mesangial cells. (**J**) Representative confocal images of Smad2/3. **K**) YAP intracellular localization in mesangial cells stimulated with FAM. Scale bar, 10 μm. (**L**) Representative immunofluorescence images of fibrotic-like mesangial. α-SMA (red) and DAPI (blue). Scale bar, 10 μm. (**M**) Schematic of FAM and mesangial lineage interactions in the DKD fibrotic niche. ** *p* < 0.01

To further investigate function and mechanism of FAM and mesangial cell in kidney fibrotic niche. We analyzed the intercellular ligand receptor and the signaling pathway of FAM and mesangial cell interaction. The results of the network diagram and circle diagram presented multiple fibrosis-related pathways (Fig. 4A and Fig. S9). The TGF-β_1_/TGF-β_1_R, Nrp1/VEGF, Nrp2/VEGF, and Notch/Jag2 pathways exhibited significant activation, especially the TGF-β_1_ signal pathway (Fig. 4B). qRT-PCR analysis showed that the expression of fibrotic factor TGF-β_1_ in macrophages of the DKD group was significantly higher than that in the control group (Fig. 4E). Nuclear transcription factor Smad2/3 and fibrosis index Col4a5 were labeled using immunofluorescence double staining in DKD kidney tissue in *vivo*. The expression of Smad2/3 in myofibroblast nuclei increased, and collagen fibers were deposited in DKD kidney tissues at significantly higher levels relative to those in the control group (Fig. 4C). YAP is the key transcription co-factor in Hippo pathway and is involved in the pathogenesis of various diseases. As DKD progresses, continual cell lineage interactions induced YAP and nuclei expression increased and colocalized with α-SMA, which widened the DKD renal fibrotic niche (Fig. 4D). The results of lysis extraction of renal tissue protein and RNA showed that the levels of YAP and Smad2/3 increased significantly with the progression of DKD (Fig. 4F and G). These results suggest that Smad2/3 and YAP nuclear translocation are involved in DKD fibrosis progression.

We stimulated macrophages, polarized them in a high-glucose environment, and cocultured them with mesangial cells for 48 h to simulate the fibrotic niche in *vitro* to clarify the synergistic effect of Smad2/3 and YAP in promoting renal fibrosis (Fig. 4H). To corroborate our in vivo findings, the expression of fibrotic proteins driven by YAP and Smad2/3 binding in mesangial cells after FAM stimulation was analyzed by Co-immunoprecipitation (Fig. 4I). After 48 h of the coculture of FAM and mesangial cells, the nuclear translocation of Smad2/3 and YAP was observed using confocal microscopy. Levels of α-SMA and Col4a5 in the cytoplasm significantly increased, and mesangial cells exhibited morphological transformation into myofibroblasts (Fig. 4J and L and Fig. S10). In order to prove YAP’s role in the DKD progression, we used the YAP inhibitor Veteporfin to intervene and found that Veteporfin could reduce DKD renal fibrosis via inhibit YAP (Fig. S11). These results indicate that FAM induce mesangial-to-myofibroblast differentiation and promote the enlargement of the DKD fibrotic niche by activating the TGF-β_1_/Smad2/3/YAP signal axis (Fig. 4M).

### MSC-sEV attenuates renal fibrosis by inhibited YAP in DKD model

**Fig. 5:**
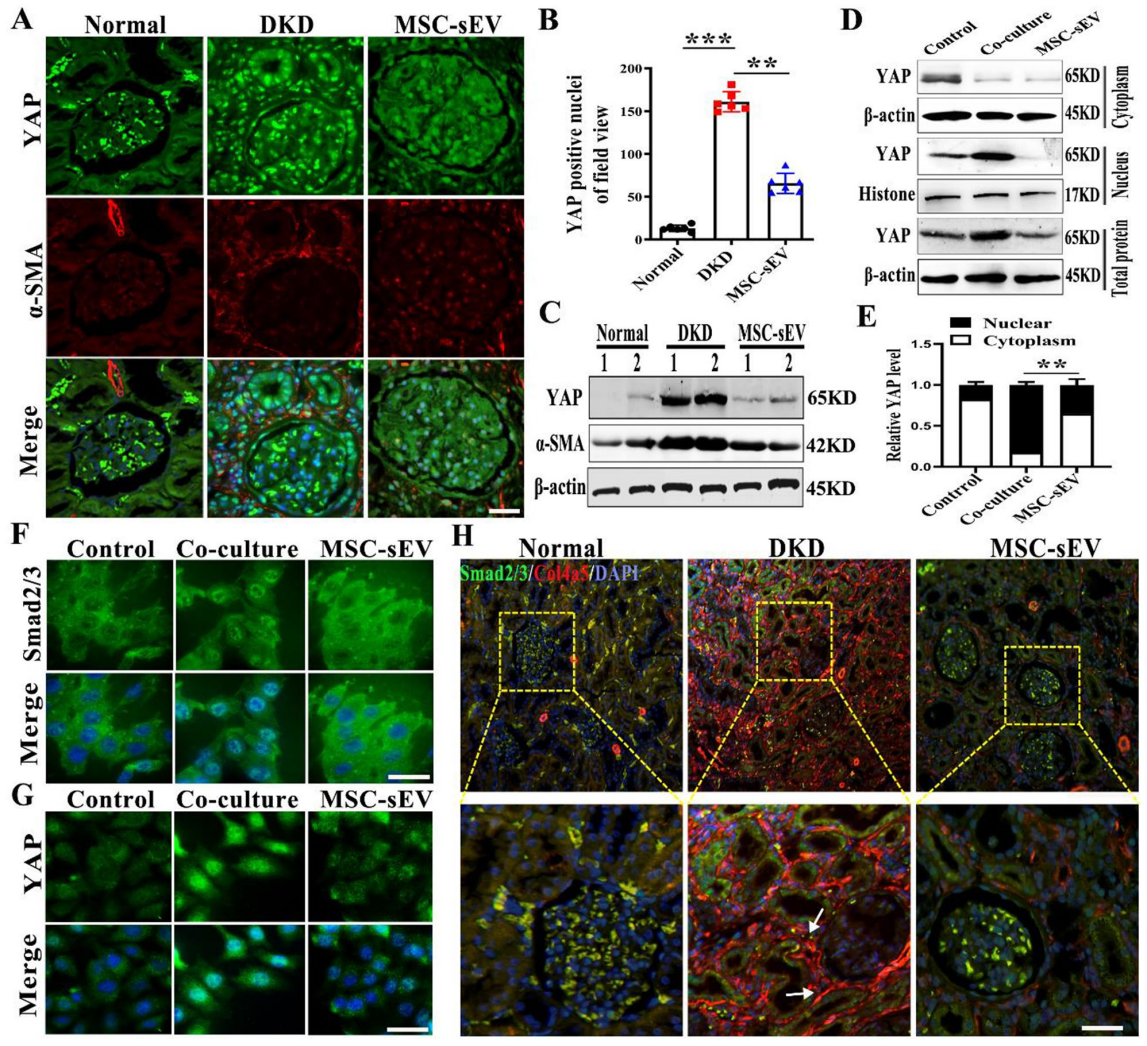
MSC-sEV inhibit YAP signaling pathway. (**A**) Representative confocal images of DKD kidney with MSC-sEV treatment. YAP (green), α-SMA (red), and DAPI (blue). Scale bar, 50 μm. (**B**) Quantification of YAP-positive nuclei per field of view in MSC-sEV group (*n* = 6). (**C**) Western blot analyses of YAP and α-SMA expression in DKD renal after MSC-sEV intervention (*n* = 2). (**D**) The expression of YAP in the cytoplasm and nucleus in mesangial cells after MSC-sEV treatment was detected by Western blot. (**E**) Statistical analysis of the expression of YAP in cytoplasm and nucleus with MSC-sEV intervention. (**F**) Representative confocal images of intracellular translocation of Smad2/3 and **G**) YAP after MSC-sEV intervention. Smad2/3 (green), YAP (green), and DAPI (blue). Scale bar, 20 μm. (**H**) Representative confocal images of DKD kidney with MSC-sEV treatment. Smad2/3 (green), Col4a5 (red), and DAPI (blue). Scale bar, 50 μm. ** *p* < 0.01, *** *p* < 0.001

To further study the therapeutic mechanism of MSC-sEV and account for the critical role of YAP in the development of kidney diseases [22, 23], we investigated whether the injection of MSC-sEV played a key role in regulating YAP. The double immunofluorescent staining and western blot showed that YAP expression in the kidneys of 24 weeks DKD rats was inhibited by MSC-sEV treatment and a decreased expression of α-SMA (Fig. 5A and C). Statistical analysis revealed that MSC-sEV treatment significantly downregulated the number of YAP positive cells (Fig. 5B). In *vitro*, YAP in the cytoplasm and nucleus was significantly reduced by extracting the cytoplasmic and nuclear proteins of mesangial after MSC-sEV treatment, and the total amount of YAP was significantly reduced (Fig. 5D and E). The confocal microscopy image was used to observe the intracellular distribution of YAP and Smad2/3 after MSC-sEV intervention. The images appeared a marked decrease in cytoplasmic retention and nuclear protein expression and a reversal of mesangial morphological changes (Fig. 5F and G). In *vitro*, MSC-sEV inhibited fibrosis like transformation of mesangial cells (Fig. S12). Double immunofluorescence staining showed that MSC-sEV inhibited the expression of Smad2/3 in DKD kidneys and downregulated the fibrosis index Col4a5 (Fig. 5H). Therefore, MSC-sEV alleviated DKD fibrosis via inhibiting YAP.

### MSC-sEV delivers CK1δ/β-TRCP mediates YAP ubiquitination and degradation

**Fig. 6:**
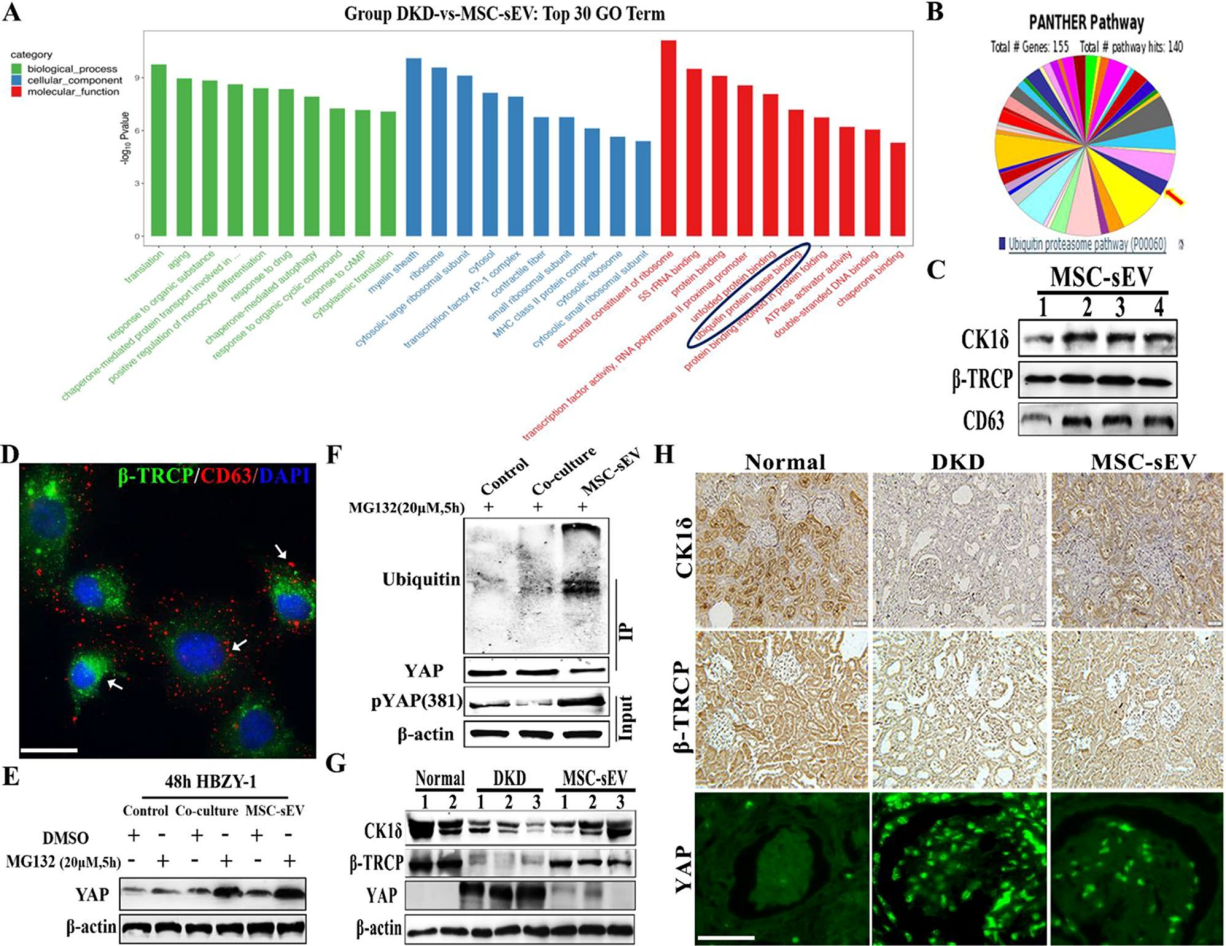
MSC-sEV derived CK1 δ and β-TRCP to promote YAP degradation. (**A**) Transcriptome analysis changes of biological process, molecular function, and cellular components in renal tissue of DKD and MSC-sEV groups. (**B**) LC-MS/MS analysis of the protein composition of MSC-sEV. (**C**) Western blot for CK1δ and β-TRCP proteins in MSC-sEV (*n* = 4). (**D**) Internalization of MSC-sEV in mesangial cells was observed using confocal microscopy. CD63 (Red). Scale bar, 20 μm. (**E**) Western blot for CK1δ and β-TRCP proteins in the kidneys of DKD rats treated with MSC-sEV (*n* = 3). (**F**) Representative immunohistochemistry and confocal images of CK1δ, β-TRCP, and YAP (green) with MSC-sEV treatment. Scale bar, 100 μm. (**G**) Co-immunoprecipitation to determine ubiquitin bound to YAP protein after MSC-sEV treatment. (**H**) Mesangial cells were stimulated by FAM, treated with MSC-sEV, pretreated with 20 µM MG132 for 5 h, and the protein level of YAP was detected by western blot

Next, we investigated the underlying mechanism in which MSC-sEV ameliorated DKD fibrosis by inhibiting YAP. We found changes of ubiquitinated ligases in DKD and MSC-sEV groups by transcriptome sequencing analysis (Fig. 6A). Then, we also found that the ubiquitination–degradation proteome system existed in MSC-sEV through liquid chromatography-tandem mass spectrometry (LC-MS/MS) (Fig. 6B). Researchers have demonstrated that the kinase ubiquitin system (CK1δ/β-TRCP) mediates YAP degradation [24, 25]. The extracellular vesicle lysates were extracted, and western blot experiments confirmed that the MSC-sEV loaded CK1δ and β-TRCP (Fig. 6C). The immunostaining using CD63 as a marker of multivesicular bodies was used to confirm that MSC-sEV could be uptake by mesangial cells. The colocalization of β-TRCP and CD63 was found in cells and in the extracellular environment (Fig. 6D), indicating that the β-TRCP was enriched in extracellular vesicles and could be released and internalized into mesangial cells through MSC-sEV.

Phosphorylation at Ser127 and Ser381 sites is critical for YAP stability [26]. This helps to understand YAP phosphorylate at Ser127 and Ser381 sites increased with MSC-sEV treatment (Fig. S13). Pretreated mesangial cells with the proteasome inhibitor MG132 (20 µM) and stimulated them with FAM in the presence of MSC-sEV, the result showed that the downregulation of YAP by MSC-sEV was restored by adding MG132 (Fig. 6E). Furthermore, co-immunoprecipitation (Co-IP) results confirmed that MSC-sEV significantly increased the ubiquitinated modification of YAP (Fig. 6F). Confocal microscopy results indicated that MSC-sEV inhibited the expression of YAP/Smad23 in the nucleus (Fig. S14). The expression of CK1δ and β-TRCP decreased and YAP was highly expressed in DKD in *vivo*. MSC-sEV increased the levels of CK1δ and β-TRCP and inhibited YAP, as indicated by immunohistochemical results (Fig. 6G and H). The levels of CK1δ and β-TRCP were similar after MSC-sEV treatment at the mRNA and tissue levels (Fig. S15). These results indicate that MSC-sEV attenuated renal fibrosis through CK1δ and β-TRCP mediate YAP ubiquitination and degradation.

### Knockdown of CK1δ/β-TRCP reduces the anti-fibrotic effect of MSC-sEV

**Fig. 7:**
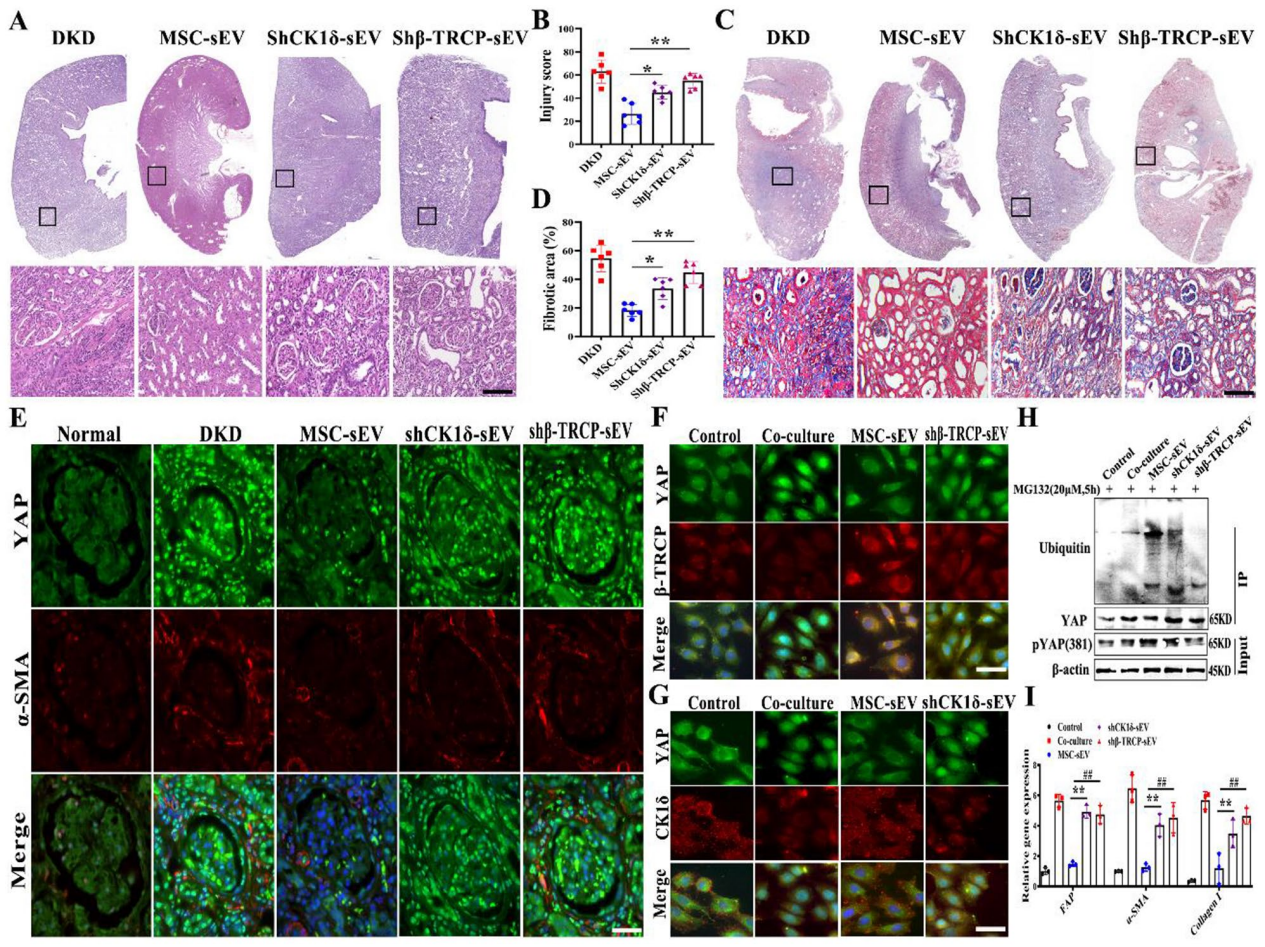
CK1δ and β-TRCP knockdown attenuates the anti-fibrotic effect of MSC-sEV. (**A**) Representative images of H&E staining (*n* = 6). The bottom image is a magnified image of the corresponding region. Scale bars, 1 mm (top) and 200 μm (bottom). (**B**) Quantification of renal injury based on H&E staining (*n* = 6). (**C**) Representative images of Masson trichrome staining on sagittal sections (*n* = 6). The bottom image is a magnified image of the corresponding region. Scale bars, 1 mm (top) and 200 μm (bottom). (**D**) Quantification of renal fibrotic area based on Sirius red staining (*n* = 6). (**E**) Representative confocal images of DKD fibrotic niche after shCK1δ-sEV and shβ-TRCP-sEV treatment. YAP (green), α-SMA (red), and DAPI (blue). Scale bar, 50 μm. (**F**) Representative confocal images of nuclear and cytoplasm localization in mesangial cells with shβ-TRCP-sEV treatment. YAP (green), β-TRCP (red), and DAPI (blue). Scale bar, 20 μm. (**G**) Representative confocal images of intracellular translocation after shCK1δ-sEV intervention. YAP (green), CK1δ (red), and DAPI (blue). Scale bar, 20 μm. (**H**) Co-immunoprecipitation determined the level of YAP ubiquitination after shCK1δ-sEV and shβ-TRCP-sEV treatment. (**I**) Statistical analysis of the expression of fibrosis-related indicators with shCK1δ-sEV and shβ-TRCP-sEV intervention. * *p* < 0.05, ** *p* < 0.01, # *p* < 0.05, ## *p* < 0.01

To evaluate whether CK1δ and β-TRCP is effective for MSC-sEV to repair DKD renal fibrosis by regulating YAP, we knocked down CK1δ and β-TRCP in MSC using adenovirus-mediated shRNA transfection. The extraction of shCK1δ-sEV and shβ-TRCP-sEV by ultracentrifugation, qRT-PCR analysis of knockdown efficiency, and CK1δ and β-TRCP in MSC-sEV were significantly downregulated by 60% after interference (Fig. S16). We then analyzed the antifibrotic effects of MSC-sEV after the knockdown of CK1δ and β-TRCP. As expected, as indicated by a histological analysis of DKD kidney sections, significant tubulointerstitial damage, including tubular atrophy, patchy infiltrates of leukocytes, and glomerular basement membrane thickening, all of which were markedly aggravated by shCK1δ-sEV and shβ-TRCP-sEV treatment (Fig. 7A and B). Additionally, the Masson staining results showed that numerous collagen fibers were deposited in the renal interstitium in the shCK1δ-sEV and shβ-TRCP-sEV groups, and the antifibrotic effects were significantly weakened (Fig. 7C and D). In the DKD rat model, the expression and nuclear localization of YAP and α-SMA were significantly increased with shCK1δ-sEV and shβ-TRCP-sEV treatment (Fig. 7E). shCK1δ-sEV and shβ-TRCP-sEV had impaired ability to inhibit YAP expression in DKD when compared with MSC-sEV.

We also found that shCK1δ-sEV and shβ-TRCP-sEV could not increase CK1δ or β-TRCP expression in FAM-stimulated mesangial cells in *vitro*(Fig. S17). Furthermore, the nuclear protein YAP expression was significantly increased in the shCK1δ-sEV and shβ-TRCP-sEV groups, indicating that the CK1δ and β-TRCP delivered by MSC-sEV play a critical role within the cells (Fig. 7F and G). The results of co-immunoprecipitation presented that the amount of ubiquitin in FAM-stimulated mesangial cells in the shCK1δ-sEV and shβ-TRCP-sEV groups were less than MSC-sEV group (Fig. 7H). In the coculture environment, the expression of fibrosis-related proteins (FAP, α-SMA, Collagen I) was increased in the shCK1δ-sEV and shβ-TRCP-sEV groups, and the antifibrotic ability disappeared after knockdown (Fig. 7I). These results indicate that the CK1δ/β-TRCP kinase ubiquitin system as the key molecules in MSC-sEV mediated YAP degradation to inhibit DKD fibrotic niche.

## Discussion

In this study, we have reported an approach based on single-cell RNA sequencing to analyze MSC-sEV administration and evaluate the feasibility and effectiveness of MSC-sEV for DKD. We have found that fibrosis-associated macrophages and mesangial cells interact to accelerate fibrosis in DKD, and MSC-sEV regulate YAP protein stability by delivering the kinase ubiquitin system, producing a significant antifibrotic effect. Our findings indicate that MSC-sEV may constitute an effective nanotherapeutic for treating DKD fibrosis.

Our main goal is to focus on early treatment and prevention of DKD injury and inhibition of renal interstitial fibrosis [27]. Stem cell-derived noncell therapeutic components are expected to be an ideal approach for early intervention in DKD [28]. Small extracellular vesicles, one of nanovesicles produced by some stem cells, have aroused interests in regenerative medicine. They facilitate cell-cell communication and play a biological role by transferring active molecules such as DNA, RNA, protein and lipid [29]. Our previous studies have confirmed that MSC-sEV can significantly reduce blood glucose levels in type 2 diabetic rats, promote insulin secretion, accelerate liver glycogen synthesis [30], and inhibit renal interstitial fibrosis in a unilateral ureteral obstruction rat model [11]. In addition, our research evidence shows that MSC-sEV intervention can improve renal function and reduce renal damage in the DKD model. Other study has indicated that markedly high levels of monocyte-derived macrophages and changes in polarization promote the progression of renal interstitial fibrosis [31]. Furthermore, we first isolated single renal cell suspension using scRNA-seq and spatial mapping. Then, we elucidated the fibrotic niche of DKD rats and identified pathogenic subpopulations of TGF-β_1_^+^Arg1^+^ macrophages, Actin^+^ and Col4a5^+^ mesangial cells. In *vivo* and in *vitro* experiments demonstrated that fibrosis-associated macrophages promoted mesangial-to-myofibroblast differentiation by activating the TGF-β_1_/Smad2/3/YAP signal axis and collagen, and increased α-SMA expression, which further slowed DKD fibrosis progression. We determined a complex and profibrotic interaction between multiple fibrosis-associated cell lineages.

To verify the mechanism and efficacy of MSC-sEV against DKD fibrosis, here, our findings show that MSC-sEV treatment promoted the upregulation of YAP phosphorylated (ser381 and ser127) and decreased the overall YAP protein level. Nevertheless, the expression and activity of YAP are controlled by its cytoplasmic retention and the ubiquitination degradation in the Hippo pathway [24, 32]. The kinase ubiquitin system regulates the stability of YAP proteins, and results from transcriptome sequencing and MSC-sEV mass spectrometry indicated that the activity of this system in DKD was significantly reduced. We also confirmed that CK1δ and β-TRCP, as the main components regulating YAP degradation, were enriched in MSC-sEV. The administration of MSC-sEV can target the damaged kidney tissues and increase the expression of CK1δ and β-TRCP to promote YAP ubiquitination. The antifibrotic efficacy of MSC-sEV was decreased significantly while CK1δ and β-TRCP were knocked down, indicating the antifibrosis of YAP degradation via MSC-sEV transported CK1δ and by β-TRCP.

**Fig. 8:**
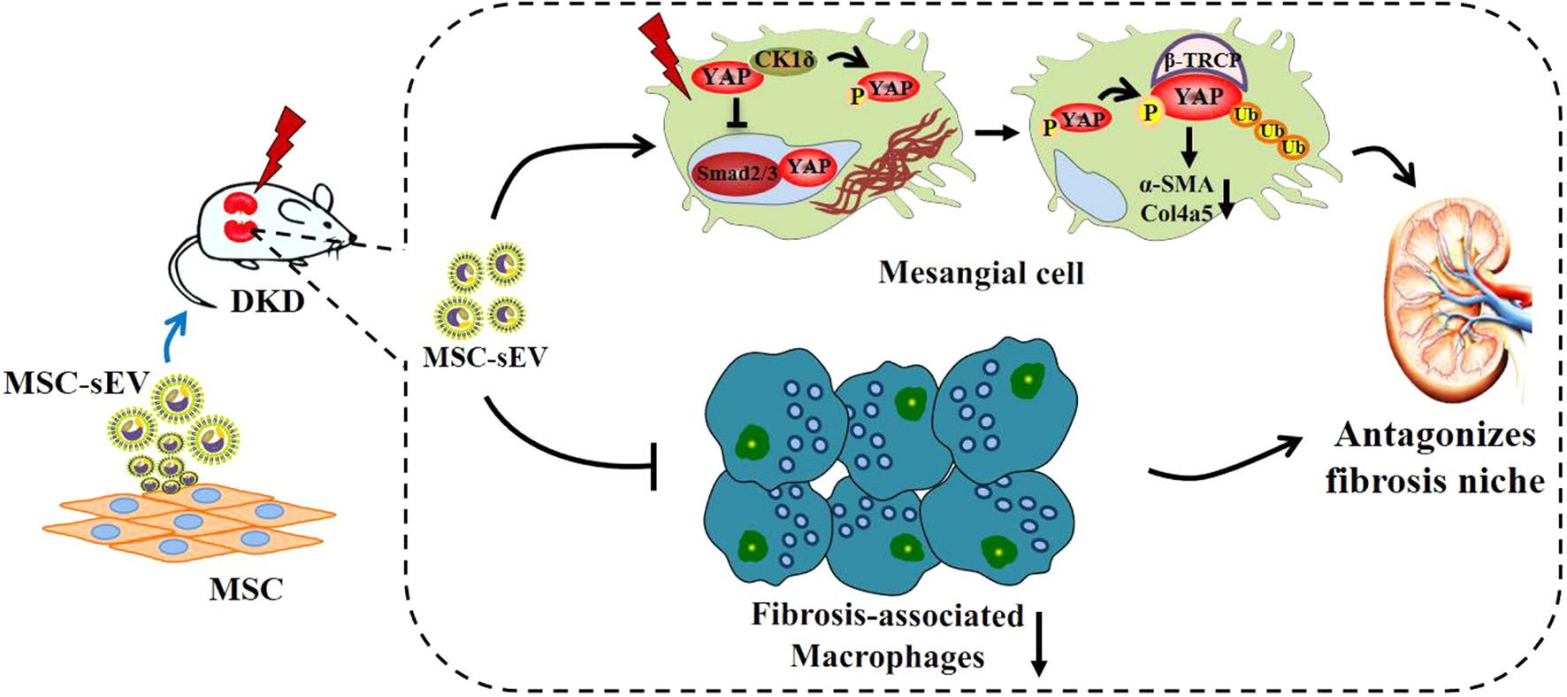
Schematic illustration: fibrosis-associated macrophages promote mesangial-to-myofibroblast differentiation by activating the TGF-β_1_/Smad2/3/YAP axis, while the MSC-sEV antagonizes the DKD fibrosis niche by CK1δ/β-TRCP-mediated YAP degradation

Together, single-cell RNA sequencing reveals that TGF-β_1_^+^Arg1^+^ fibrosis-associated macrophages appeare in DKD and promote mesangial-to-myofibroblast differentiation by TGF-β_1_/Smad2/3/YAP signaling axis and accelerate DKD fibrotic niche formation. MSC-sEV loaded CK1δ and β-TRCP can inhibit renal fibrosis by promoting YAP ubiquitination and degradation. MSC-sEV may be used as a nanotherapeutic drug that targets renal tissue to inhibit YAP activity and improve renal fibrosis, providing a novel and effective therapeutic strategy for antifibrotic therapy. Our findings indicate that MSC-sEV has a very important prospect in the prevention and treatment of DKD and in achieving effective antifibrosis (Fig. 8).

## Materials and methods

### Experimental reagents

#### Animals

Male 8-week-old Sprague–Dawley rats weighing 150 g were purchased from Jiangsu Laboratory Animal Center to prepare animal models. The rats were housed in a specific pathogen-free environment at the Animal Center of Jiangsu University at the optimal temperature with a 12 h light/12 h dark cycle. DKD model by high-fat diet combined with streptozotocin (STZ, 35 mg/kg) injected into tail vein. All animal experiments were performed in strict accordance with the National Institutes of Health Guide for the Care and Use of Laboratory Animals guidelines and were approved by the Ethics Committee of Jiangsu University (2,022,264, Jiangsu, China).

#### Isolation and identification of MSC-sEV

MSC-sEV were isolated and purified. Briefly, the conditioned medium was collected and centrifuged at 1,000 g for 20 min to remove cell debris, followed by centrifugation at 2,000 g for 20 min and 10,000 g for 20 min. The supernatant was collected and concentrated using 100 KDa molecular weight cut off (MWCO) (Millipore) at 1,000 g for 30 min. The concentrated supernatant was loaded 5 mL and then ultracentrifuged at 100,000 g for 60 min (optimal-90 K; Beckman Coulter). The exosome-enriched fraction was diluted with PBS, and then centrifuged thrice at 1,000 g for 30 min using 100 KDa MWCO. Finally, the purified exosomes were subjected to filtration on a 0.22-µm pore filter (Millipore) and stored at − 80 °C.

#### Histological analysis

Formalin-fixed, paraffin-embedded, 2 μm thick kidney sections were stained with H&E (Masson and Sirius Red), and their histological score was determined. A renal pathologist assessed the severity of tubulointerstitial fibrosis. Scoring was performed in a blinded fashion in ten consecutive fields at a magnification of 400× per section. All tests were repeated three times.

#### Immunohistochemistry and immunofluorescence

The kidney was fixed in 4% paraformaldehyde, embedded in paraffin and then cut into 2 μm-thick sections. Immunohistochemistry was performed to assess renal injury and fibrosis. In addition, immunofluorescence was performed to detect the colocalization of fibrotic proteins in mesangial cells and to assess renal inflammatory infiltration of macrophages observed by confocal microscopy. Immunofluorescence co-staining was performed using an immunohistochemistry kit (Boster, China). Sections were stained with the following antibodies: monoclonal rabbit anti-YAP (14,074 S, CST, USA), monoclonal mouse anti-α-SMA (19,245 S, CST, USA), monoclonal rabbit anti-F4/80 (70076T, CST, USA), monoclonal rabbit anti-CK1δ (12,417 S, CST, USA), and monoclonal rabbit anti-β-TRCP (4394 S, CST, USA). Positive cells were counted in the renal interstitial on five nonoverlapping view fields at 400× magnification.

#### Western blotting

Kidney tissues were harvested from DKD rats in different groups, lysed with radioimmunoprecipitation assay (RIPA) buffer (Sigma, St. Louis), and supplemented with multiple protease inhibitors (Invitrogen, USA). We separated 100-µg protein samples by 12% SDS-PAGE. After semidry transfer, nonspecific binding sites of the nitrocellulose membrane were blocked with 5% nonfat milk in Tris-buffered saline. Subsequently, the membrane was incubated with the following primary antibodies: monoclonal rabbit anti-α-SMA (19,245 S, CST, USA), monoclonal rabbit anti-TGF-β_1_ (3709 S, CST, USA), monoclonal rabbit anti-YAP (14,074 S, CST, USA), monoclonal rabbit anti-phospho-YAP (ser381) (13,008 S; CST, USA), monoclonal rabbit anti-CK1δ (12,417 S, CST, USA), and monoclonal rabbit anti-β-TRCP (4394 S, CST, USA). Subsequently, the conjugated antibodies incubated with secondary horseradish peroxidase (HRP) that were used were anti-mouse immunoglobulin G (IgG) and anti-rabbit IgG (Abcam) for 2 h at room temperature. Blots were analyzed with the enhanced chemiluminescence (ECL) system and captured on autoradiographic films. Glyceraldehyde 3-phosphate dehydrogenase (GADPH) and β-actin were blotted on the same membrane as the loading controls.

#### Macrophages and mesangial cell coculture

First, the macrophages were cultured in a high-glucose environment for 48 h, and primary mesangial (20,000 cells) were seeded into the cell coculture plate with macrophages (15,000 cells) from individual DKD rats (*n* = 5) in Gibco Dulbecco’s Modified Eagle Medium. All growth factor supplements were removed, and cells were cultured for 72 h in mesangial basal media. Mesangial cells were fixed in 4% paraformaldehyde for 30 min, permeabilized with 0.3% Triton phosphate-buffered saline (PBS) for 5 min, blocked with 10% serum in PBS for 30 min, and finally subject to primary antibody incubation (mouse anti-α-SMA and rabbit anti-Collagen 1) for 12 h. Next, cells were washed in 0.1% Triton PBS followed by the addition of fluorescently conjugated secondary antibodies (1:500 dilutions) for 2 h. Cells were mounted with the nuclear dye DAPI, and images were taken using a confocal microscope GE.

#### Preparation of single cell suspension

Euthanized rats were perfused with chilled 1x PBS via the left heart. Kidneys were harvested, minced into approximately 1 mm^3^ cubes and digested using Multi Tissue dissociation kit (Miltenyi, 130-110-201). Up to 0.25 g of the tissue was digested with 50 ul of Enzyme D, 35 ul of Enzyme R and 10 ul of Enzyme A in 1 ml of RPMI and incubated for 30 min at 37 degrees. Reaction was deactivated by adding 10% FBS. The solution was then passed through a 40 μm cell strainer. After centrifugation at 1,000 RPM for 5 min, cell pellet was incubated with 1 ml of RBC lysis buffer on ice for 3mins. Single cells were washed with PBS, and the cell number were analyzed using (Countess Auto Counter) and viability (Trypan).

#### Data quality control and preprocessing

Once the gene-cell data matrix was generated, poor quality cells were excluded, such as cells with < 200 or > 3,000 unique genes expressed genes (as they are potentially cell duplets). Only genes expressed in 10 or more cells were used for further analysis. Cells were also discarded if their mitochondrial gene percentages were over 50%. The data were natural log transformed and normalized for scaling the sequencing depth to a total of 1e4 molecules per cell, followed by regressing-out the number of UMI using Seurat package. Batch effect was corrected by using remove function of edgeR.

#### Dimensionality reduction and tSNE visualization

Seurat R package (version 1.4.0.5) was used for dimensionality reduction analysis. We first identified highly variable genes across the single cells, after controlling for the relationship between average expression and dispersion. Genes were placed into 20 bins based on their average expression and removed using 0.0125 low cutoff and 0.3 high cutoff. Within each bin, a z-score of log transformed dispersion measure (variance/mean) was calculated. A z-score cutoff of 0.5 was applied to identify the highly variable genes, resulting in a total of 1,140 genes. Then we performed PCA using the variable genes as input and determined significant PCs based on the jackStraw function from the Seurat package. Statistically significant 20 PCs were selected as input for t-Distributed Stochastic Neighbor Embedding (tSNE). tSNE visualized the single cells on a two-dimensional space based on expression signatures of the variable genes, and therefore similar to PC loadings.

#### Identification of differentially expressed genes and marker genes

cell specific marker genes were identified in two stages. The first sets of differentially expressed genes (DEGs) were identified by comparing cells in a specific cluster with cells in all other clusters (Seurat package likelihood ratio test: average expression difference > 0.5 natural log with a FDR corrected *p* < 0.01). Next, cells in a specific cluster were compared to cells in every other cluster in a pairwise manner to identify a second sets of DEGs (Seurat package likelihood ratio test: average expression difference > 0.25 natural log with *p* < 0.05). Cell specific markers were identified by overlapping first and second sets of DEGs. Since different cells in the kidney share some well-known markers (transitional cells vs. intercalated cells and proximal tubule vs. novel cells), the combination of these two approaches using the lower threshold enabled us to retain the shared markers while identifying distinct markers compared to other cells.

#### Cell clustering analysis

The density-based spatial clustering algorithm (DBSCAN) was used to identify cell types on the tSNE map with an initial setting of an eps value of 0.5. Clusters were removed if their number of cells was less than 10. The remaining cells were clustered again with an eps value of 1, followed by the removal of clusters if the number of cells was less than 20. After pruning, we removed 320 cells (1.1% of our data), and 27,424 cells were used for further analysis. In a post-hoc test of the final 16 clusters, every pair was found to have more than 10 differentially expressed genes (average expression difference > 1 natural log with a FDR corrected *p* < 0.01). We used the same procedure for subclustering with modifications. Then DBSCAN was used to identify cell types on the tSNE map with an initial eps value of 0.5. Briefly, though 6 steps (①Preparation of single renal cell suspension, ②Single cell RNA sequencing: library construction and quality control, ③Data quality control and preprocessing, ④Dimensionality reduction and tSNE visualization, ⑤Identification of differentially expressed genes and marker genes, ⑥Cell clustering analysis: dimensionality reduction analysis and tSNE map showed that renal cells were divided into 12 clustering) annotated the 12 populations in Fig. 2A.

#### Statistical analysis

All experiments were performed at least three times for each group, and the statistical analyses were performed using GraphPad Prism Software (version 7). The results were presented as mean values ± standard deviation. One-way analysis of variance (ANOVA) and two-way ANOVA for multiple groups and Student’s t test for two groups were applied for statistical analysis. Survival time was analyzed using the Kaplan–Meier method and log-rank test. A *p* value < 0.05 indicated statistical significance.

### Electronic supplementary material

Below is the link to the electronic supplementary material.

Supplementary Material 1

## Data Availability

All data needed to evaluate the conclusions in the paper are present in the paper and/or the Supplementary Materials. All data in this study are available from the corresponding author upon reasonable request.
