# Supplementary material for: Single-cell RNA transcriptomic reveal the mechanism of MSC derived small extracellular vesicles against DKD fibrosis
Source: J Nanobiotechnology. 2024 Jun 18;22:339. doi: 10.1186/s12951-024-02613-2 (PMC11184851; doi:10.1186/s12951-024-02613-2)

Supplementary Materials

**Single-cell RNA transcriptomic reveal the mechanism of MSC derived small extracellular vesicles against DKD fibrosis**

Cheng Ji^1,2#^, Jiahui Zhang^2#^, Hui Shi^2^, Binghai Chen^3^, Wenrong Xu^2^, Jianhua Jin^1*^, Hui Qian^1,2,4*^.

^1^ Wujin Institute of Molecular Diagnostics and Precision Cancer Medicine of Jiangsu University, Wujin Hospital Affiliated with Jiangsu University, Chang Zhou, 213004, Jiangsu, China

^2^ Jiangsu Key Laboratory of Medical Science and Laboratory Medicine, Department of Laboratory Medicine, School of Medicine, Jiangsu University, Zhenjiang, 212013, Jiangsu, China

^3^ Institute of Translational Medicine, Jiangsu University, Department of Urology, Affiliated Hospital of Jiangsu University, Zhenjiang, 212001, Jiangsu, China

^4^ NHC Key Laboratory of Medical Embryogenesis and Developmental Molecular Biology & Shanghai Key Laboratory of Embryo and Reproduction Engineering, ShangHai, 200040, China

^#^ These authors contributed equally to this work.

^*^Corresponding author Email: [lstmmmlst@163.com;](mailto:lstmmmlst@163.com;) jianhuajin88@sina.com

This file includes:

Fig. S1. Construction of DKD rat model

Fig. S2. The biodistribution of MSC-sEV in DKD model

Fig. S3. MSC-sEV was the key component of anti-fibrotic in DKD

Fig. S4. Expression of fibrosis related proteins after MSC-sEV treatment

Fig. S5. Single cell sequencing analysis of cell community changes

Fig. S6. MSC-sEV significantly inhibited the inflammatory infiltration of neutrophils in DKD

Fig. S7. MSC-sEV induced a shift in renal macrophages

Fig. S8. MSC-sEV had an inhibitory effect on macrophages in DKD model

Fig. S9. Circle diagram of cellular communication between macrophages and mesangial cells

Fig. S10. FAM induced mesangial-to-myofibroblast differentiation

Fig. S11. Veteporfin reduced DKD fibrosis via inhibit YAP

Fig. S12. MSC-sEV inhibited fibrosis like transformation of mesangial cells

Fig. S13. MSC-sEV promoted the phosphorylation of YAP protein at Ser127 and Ser381sites

Fig. S14. MSC-sEV inhibited the expression of YAP/Smad23 in the nucleus

Fig. S15. The expression of CK1δ/β-TRCP after MSC-EVs treatment

Fig. S16. Knockdown of CK1δ/β-TRCP in MSC-EVs

Fig. S17. qRT-PCR detected the gene expression of CK1δ/β-TRCP with MSC-EVs treatment

Figure S18. Unedited gel images for figure


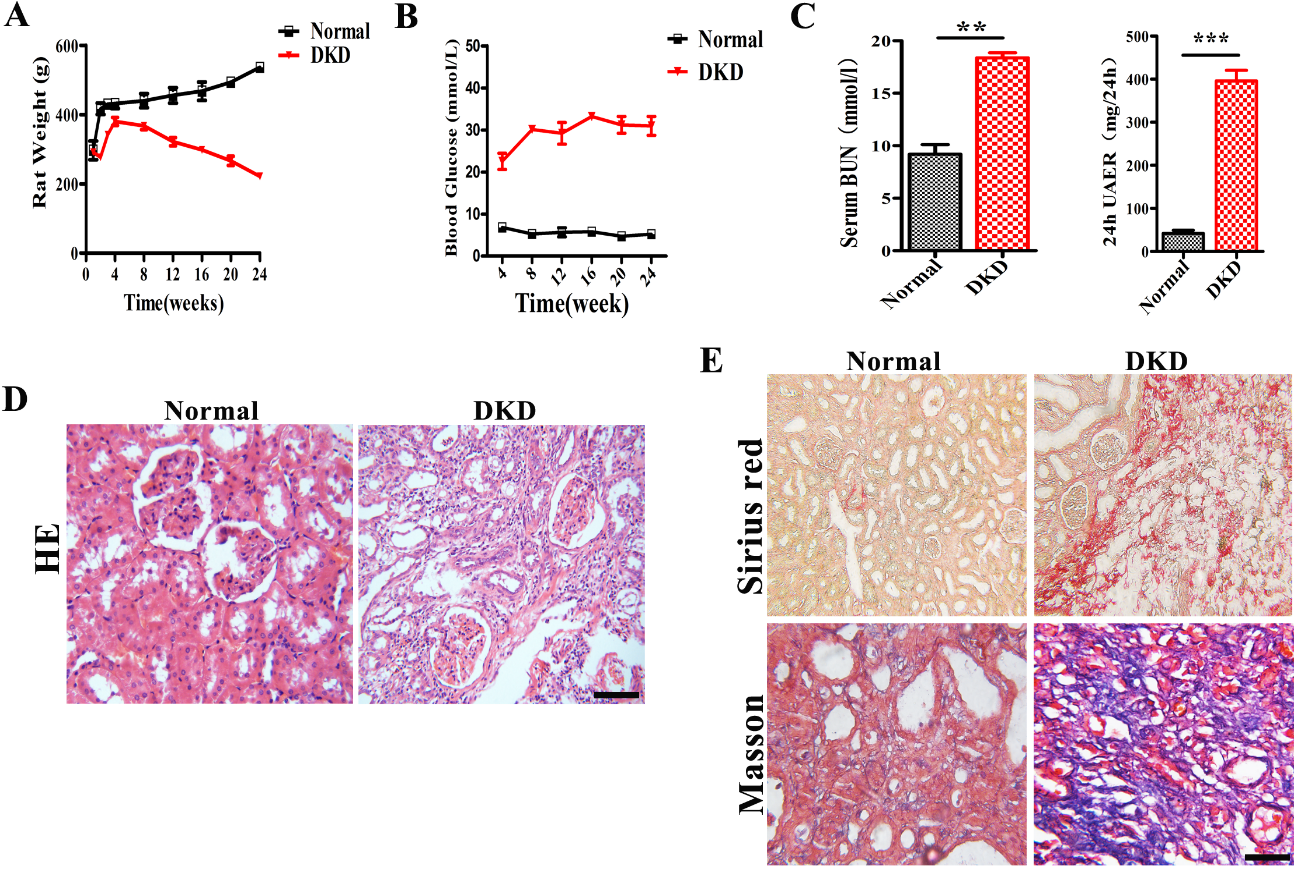


**Fig S1. Construction of DKD rat model**

**A**. Body weights of DKD rats were recorded over 24 weeks. **B**. Blood glucose monitoring in rats. **C**. Kidney functions indicators (BUN and CREA) of DKD rats were determined by blood biochemical tests. Rat blood samples were collected at 16 weeks. **D**. Analysis of DKD kidney pathological structure by HE staining. **E**. Sirius red staining and Masson staining of DKD kidney. Scale bar, 100 μm. ** *p* < 0.01, *** *p* < 0.001.


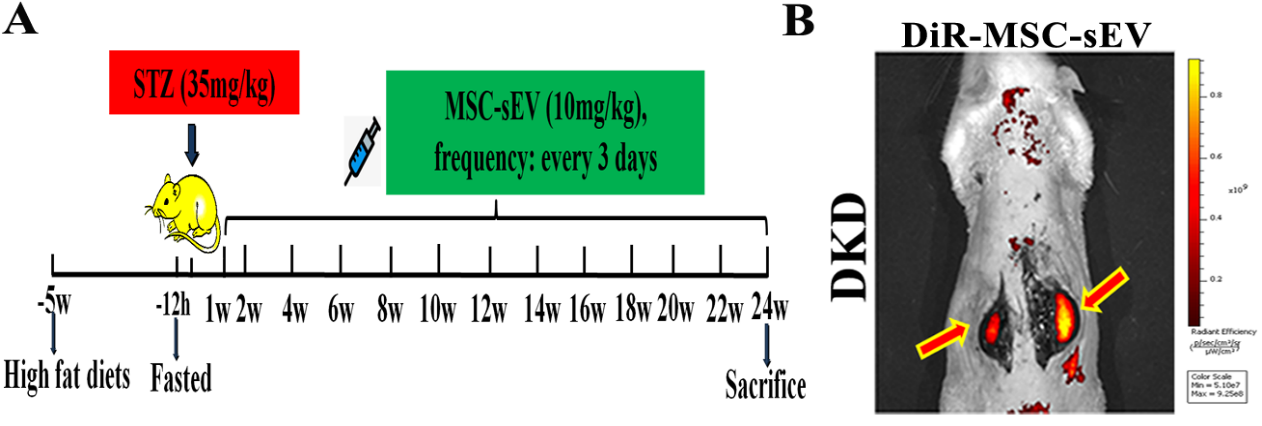


**Fig S2. The biodistribution of MSC-sEV in DKD model**

**A**. Schematic diagram of MSC-sEV (10 mg/kg of body weight; 3d/times) treatment in DKD model (n=10 per group). **B**. The distribution of CM-DIR labeled MSC-sEV injected DKD rats was detected by IVIS imaging system.


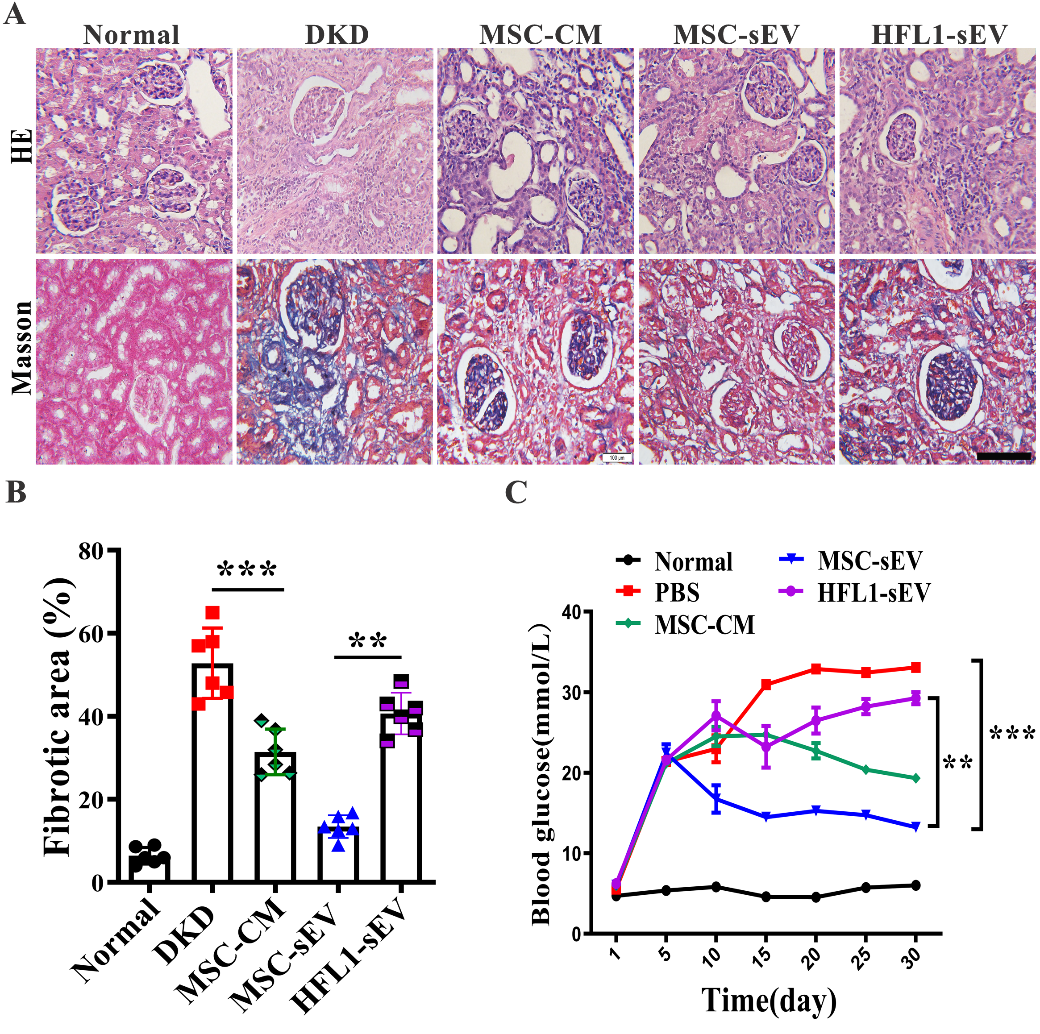


**Fig S3. MSC-sEV was the key component of anti-fibrotic in DKD**

**A**. Representative pathological images of H&E and Masson trichrome staining of DKD rats treated with PBS, MSC-CM, MSC-sEV and HFL1-EVs. Scale bar, 200 μm. **B**. The quantification of renal fibrotic area treated with PBS, MSC-CM, MSC-sEV and HFL1-EVs based on Masson trichrome staining (n = 6). **C**. The effects of PBS, MSC-CM, MSC-sEV and HFL1-EVs on the blood glucose of DKD rats were examined by glucometer. ** *P* <0.01, *** *P* <0.001.


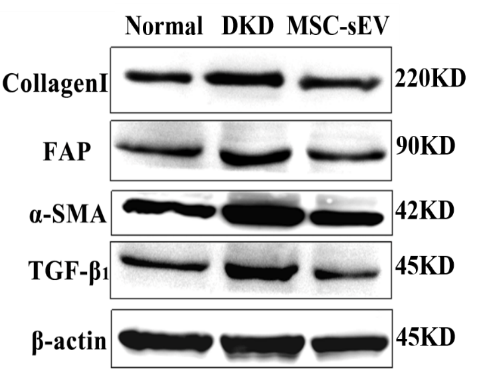


**Figure S4. Expression of fibrosis related proteins after MSC-sEV treatment**

Western blotting analysis expression of fibrosis related proteins (Collagen Ⅰ, FAP, α-SMA and TGF-β_1_) after MSC-sEV treatment in DKD renal tissue.


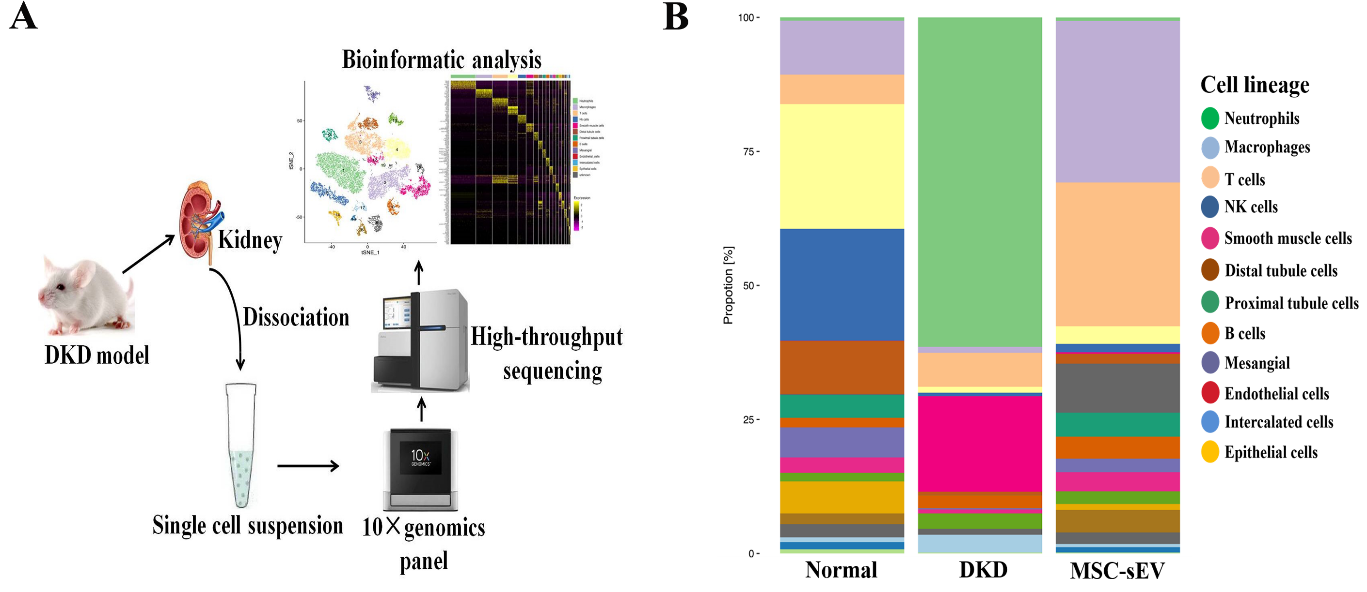


**Figure S5. Single cell sequencing analysis of cell community changes**

**A.** Overview: isolation and sc-RNA Seq of DKD renal tissue cells. **B**. The number distribution proportion of cell clusters in normal group, DKD group and MSC-sEV group.


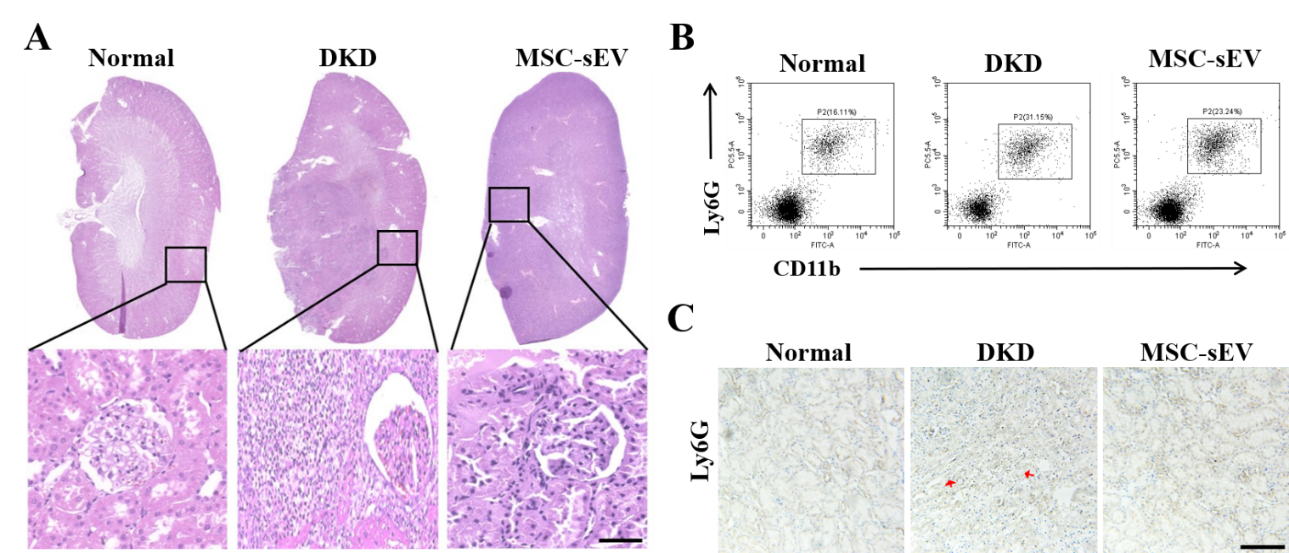


**Figure S6. MSC-sEV significantly inhibited the inflammatory infiltration of neutrophils in DKD**

**A**. Representative pathological images of H&E staining in DKD rats after MSC-sEV intervention (n =3). Scale bar, 100 μm. **B**. The expression of Ly6G in renal tissue was detected by immunohistochemical staining. **C**. The number of neutrophils in kidney homogenate was analyzed by flow cytometry. Scale bar, 200 μm.


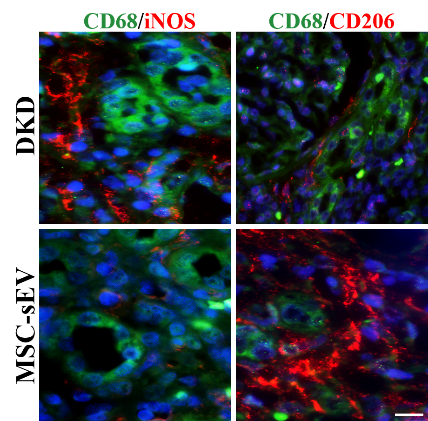


**Figure S7. MSC-sEV induced a shift in renal macrophages**

Representative confocal images of CD68^+^iNOS^+^ and CD68^+^ CD206^+^ macrophages in DKD kidney sections (n=3). The shift of macrophages was indicated by CD206^+^CD68^+^. Scale bar, 10 μm.


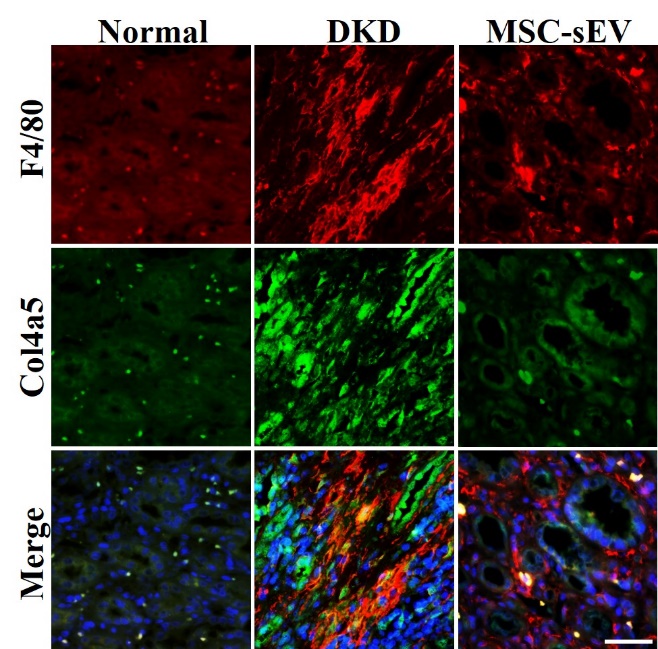


**Figure S8. MSC-sEV had an inhibitory effect on MMT cells**

Representative confocal microscopy images of F4/80 (red) and Col4a5 (green) expression with MSC-sEV treatment. Scale bar, 100 μm.


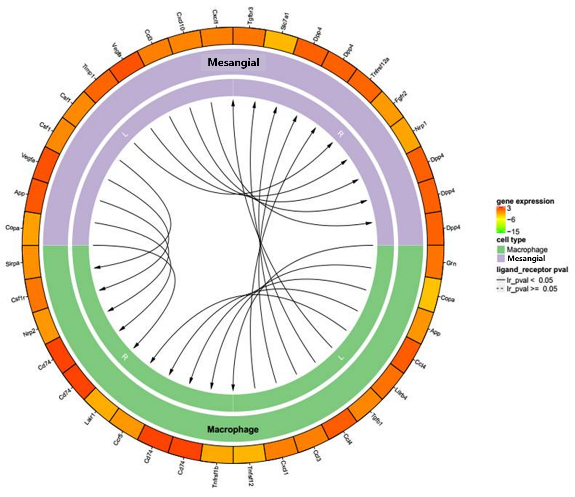


**Figure S9. Circle diagram of cellular communication between macrophages and mesangial cells**


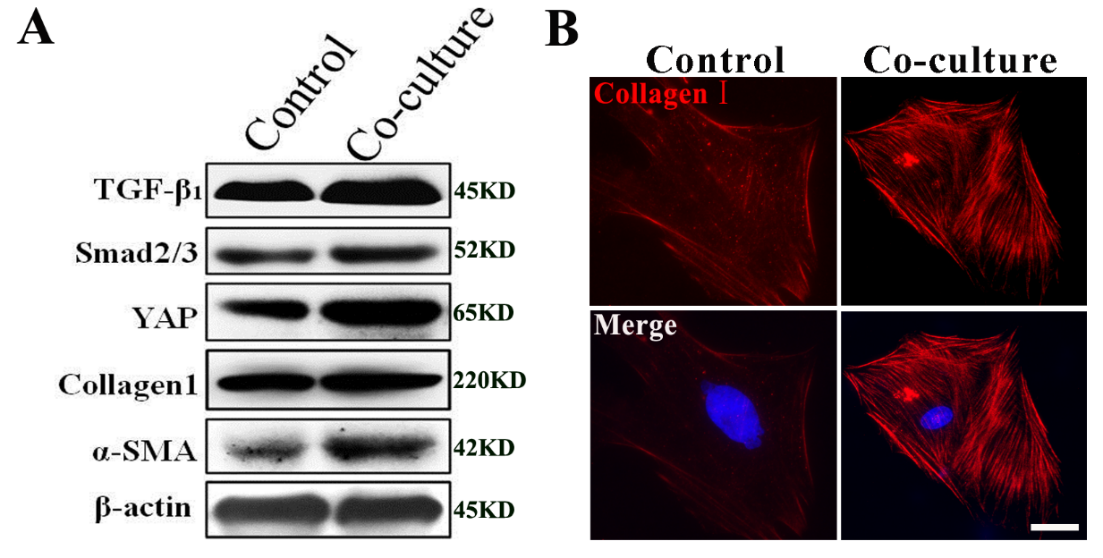


**Figure S10.** **FAM induced mesangial-to-myofibroblast differentiation**

**A**. Western blotting analysis of fibrosis-related proteins in mesangial cells stimulated with macrophages. **B**. Representative immunofluorescence images of fibrotic changes in mesangial cells, Collagen Ⅰ (red), DAPI (blue), Scale bar, 10μm.


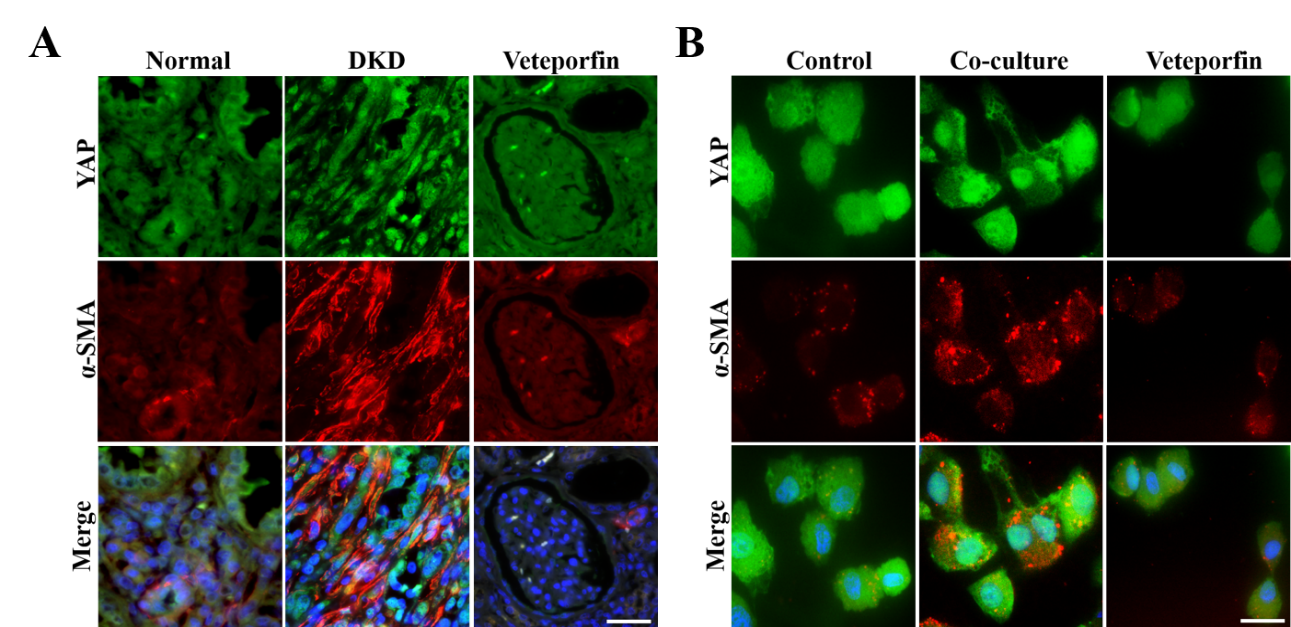


Fig. S11. Veteporfin reduced DKD fibrosis via inhibit YAP

**A.** Representative immunofluorescence images of DKD tissue with Veteporfin treatment, YAP (green), α-SMA (red), DAPI (blue). Scale bar, 50μm. **B.** Representative confocal images of YAP intracellular translocation after Veteporfin intervention. YAP (green), α-SMA (red), DAPI (blue). Scale bar, 20 μm.


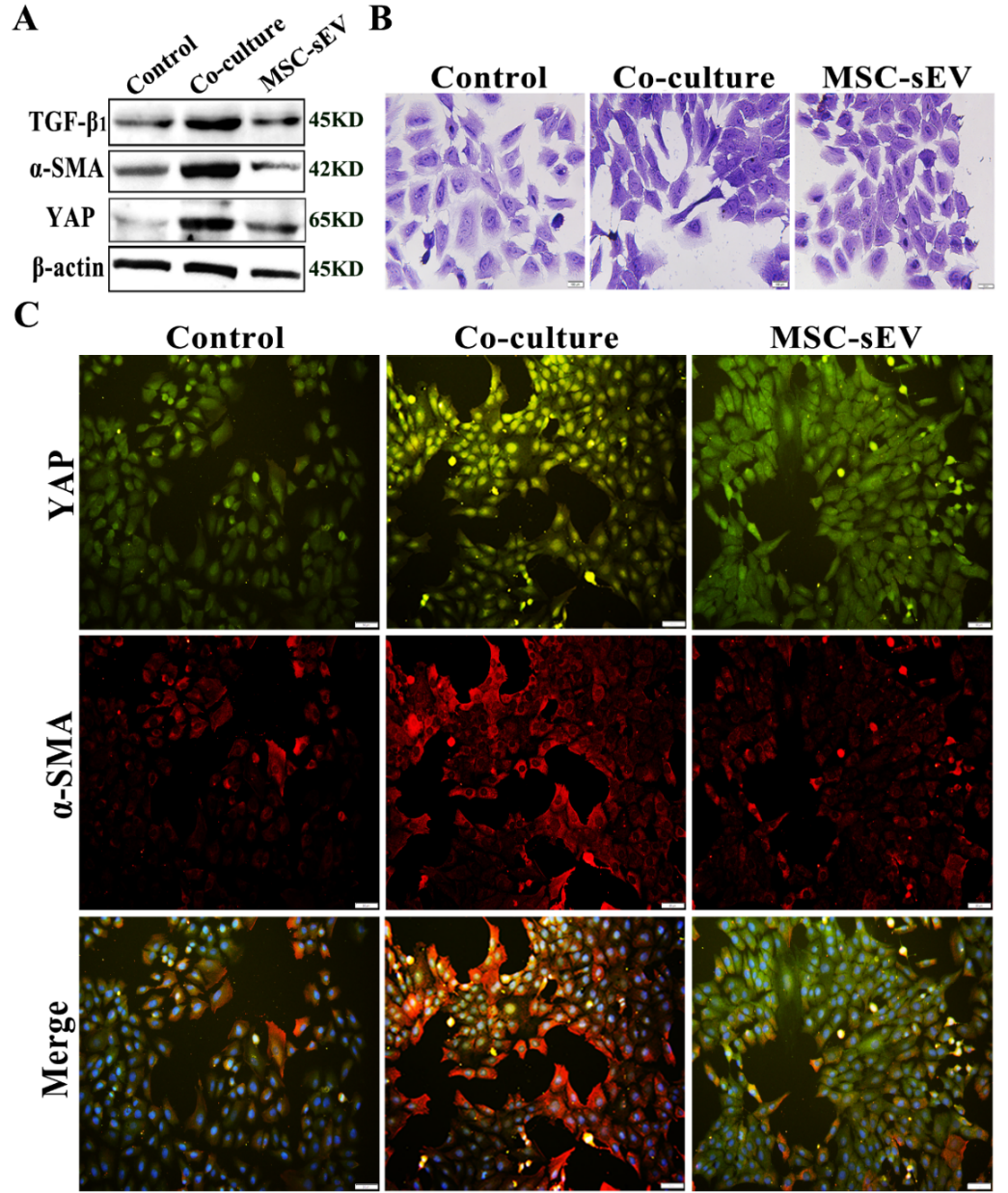


**Figure S12. MSC-sEV inhibited fibrosis like transformation of mesangial cells**

**A**. Western blotting analysis expression of fibrosis related proteins (YAP, α-SMA and TGF-β_1_) after MSC-sEV treatment in mesangial. **B**. Representative images of FAM stimulated mesangial with MSC-sEV treatment. Scale bar, 50μm. **C**. Representative immunofluorescence images of mesangial cells after MSC-sEV treatment, YAP (green), α-SMA (red), DAPI (blue). Scale bar, 50μm.


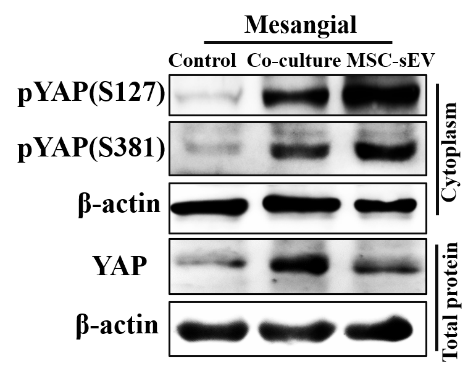


**Figure S13. MSC-sEV promoted the phosphorylation of YAP protein at Ser127 and Ser381 sites**


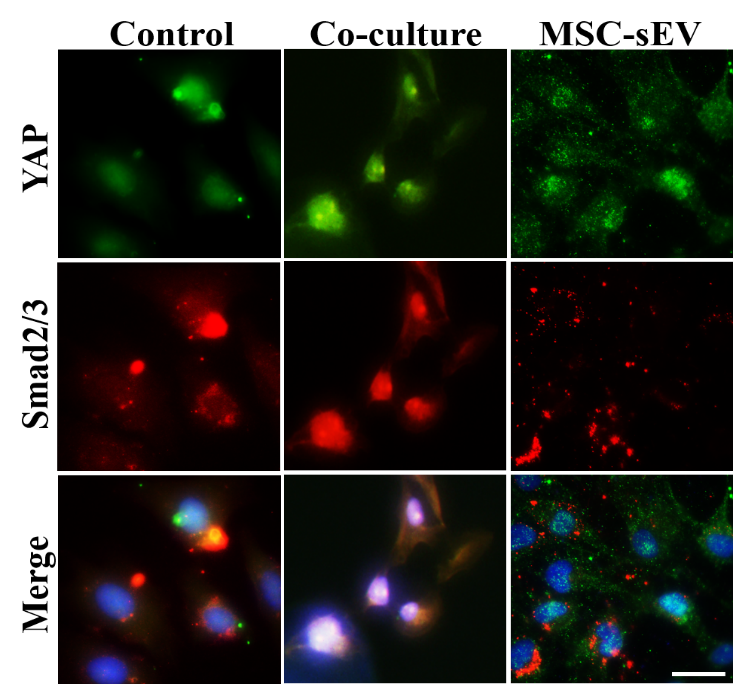


**Figure S14. MSC-sEV inhibited the expression of YAP/Smad2/3 in the nucleus**

Representative immunofluorescence images of the co-expression of YAP and Smad2/3 in the nucleus of mesangial cells after co-culture with macrophages, YAP (green), Smad2/3 (red), DAPI (blue). Scale bar, 20μm.


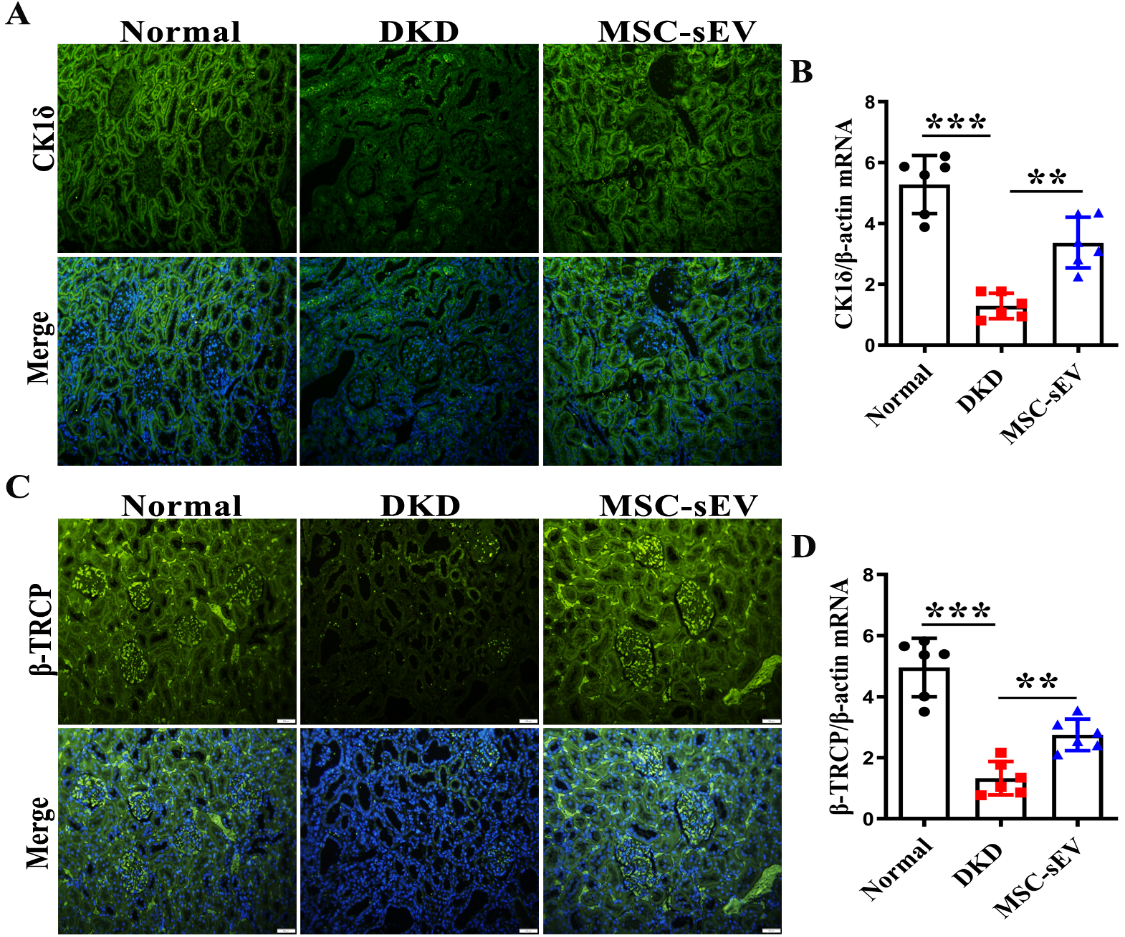


**Figure S15. The expression of CK1δ/β-TRCP after MSC-EVs treatment**

**A**. The expression of CK1δ and **C)** β-TRCP proteins after MSC-EVs intervention in kidney tissue. Scale bar, 100 μm. **B**. qRT-PCR analysis the mRNA levels of CK1δ and **D)** β-TRCP in DKD renal tissue with MSC-sEV treatment. ** *P* <0.01, *** *P* <0.001.


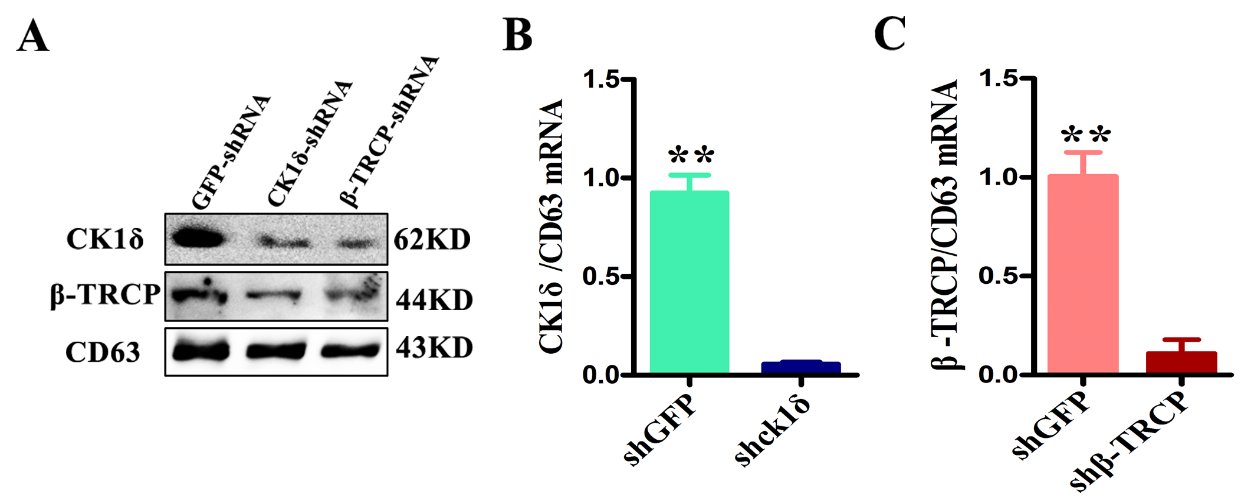


**Figure S16. Knockdown of CK1δ/β-TRCP in MSC-EVs**

**A**. Western blot detection of CK1δ/β-TRCP expression changes of MSC-EVs after adenovirus knockdown. **B**. qRT-PCR analysis of the CK1δ/β-TRCP gene level of MSC-EVs after knockdown. **C.** qRT-PCR detects the gene expression of CK1δ in mesangial cells. * *P* <0.05, ** *P* <0.01.


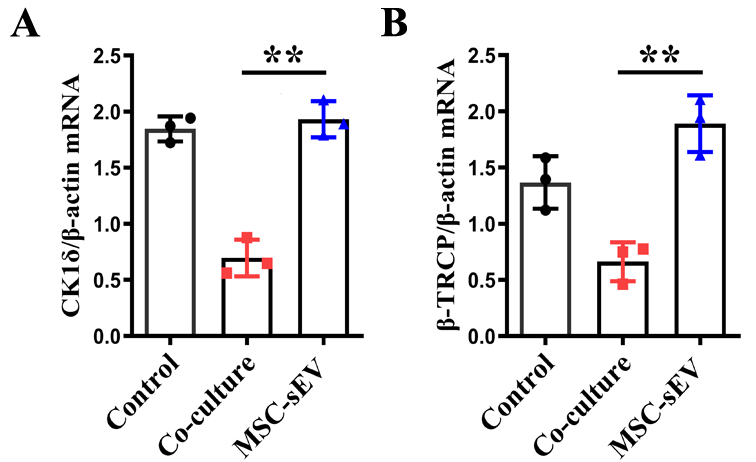


**Figure S17. qRT-PCR detected the gene expression of CK1δ/β-TRCP with MSC-EVs treatment.** * *P* <0.05, ** *P* <0.01.

**Figure S18. Unedited gel images for figure**


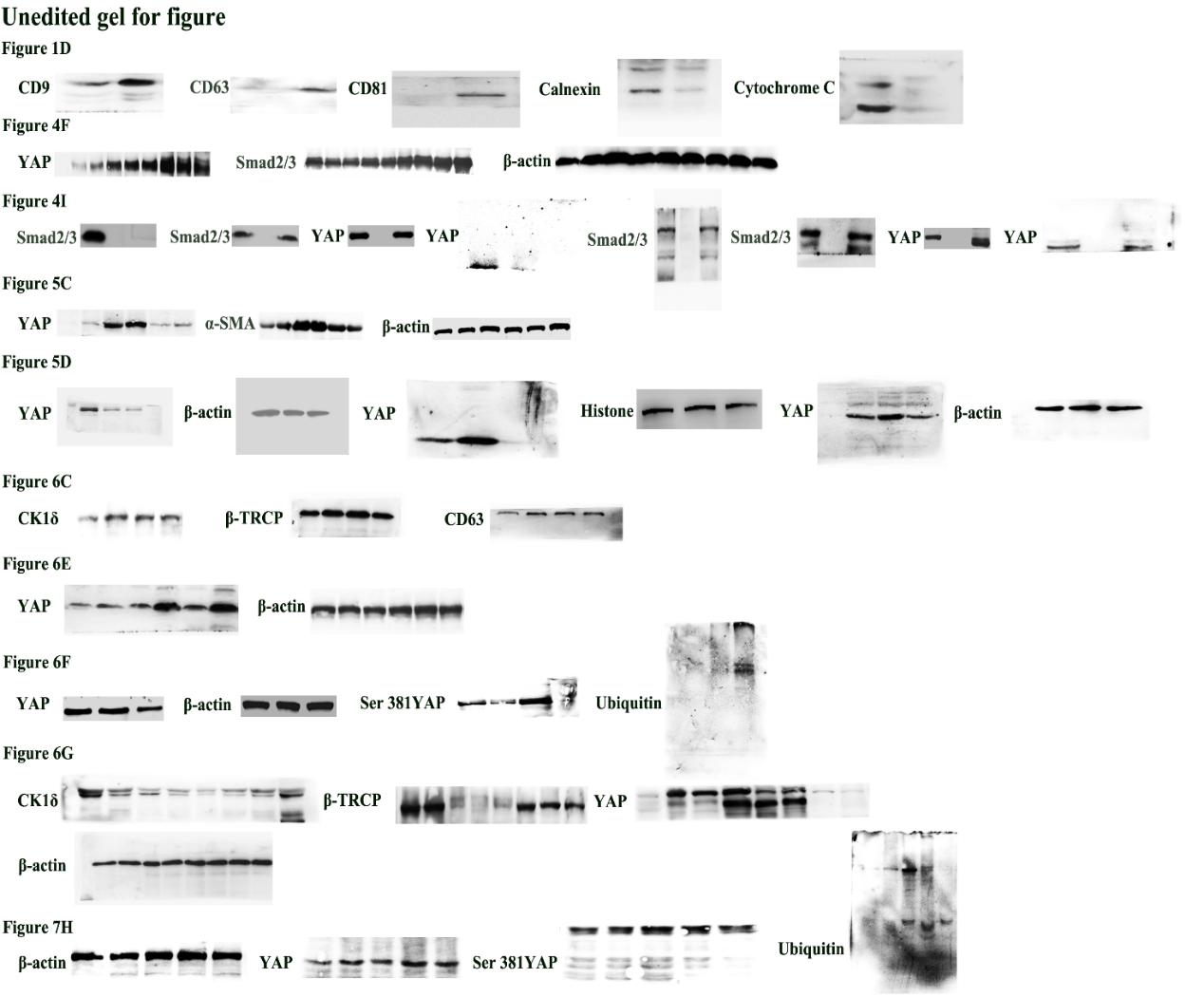

Supplement: Supplementary file 1 — Supplementary Material 1 [file 12951_2024_2613_MOESM1_ESM.docx]
